# Vestibular contributions to balance control during stair negotiation versus walking and changes with aging

**DOI:** 10.1007/s00221-025-07141-5

**Published:** 2025-08-18

**Authors:** Alexander Kern, Megan Elwood, Mike Vakula, Youngwook Kim, Eadric Bressel, Christopher J. Dakin

**Affiliations:** 1 Department of Kinesiology and Health Sciences, Utah State University, Logan, UT USA; 2 Soonchunhyang University, Asan City, South Korea

**Keywords:** Vestibular, Stairs, Walking, Galvanic vestibular stimulation, Aging

## Abstract

Falls on stairs become more likely with age, partly due to deterioration of the sensorimotor systems essential to balance. The vestibular system, critical for balance control, experiences significant deterioration losing up to 40% of its motion-sensing hair cells by age 70. Signal transmittance also appears to increasingly act like a low-pass filter with age, due to several potential mechanisms. This study aimed to explore the vestibular contributions to balance control during stair negotiation to determine if aging increases reliance on low-frequency vestibular signals similar to standing during dynamic tasks. We hypothesized that older adults would exhibit greater low-frequency vestibular stimulus correlated responses than younger adults during walking and stair negotiation. Fifteen young and fifteen older adults performed stair ascent and stair descent, and treadmill walking while receiving electric vestibular stimulation. Vestibular stimulus correlated responses were quantified by measuring coherence and gain between a 0–25 Hz random waveform electric vestibular stimulus electromyographic activity in seven hip and leg muscles. We found older adults generally exhibited greater ‘low’ frequency (0–10 Hz) coherence relative to younger adults, whereas, younger adults exhibited greater coherence at higher frequencies (10–25 Hz). These findings not only provide evidence in support our hypothesis but also invite a broad discussion of alternative interpretations that may underlie the age-related shift in how different frequency vestibular signals influence muscle activity during dynamic balance tasks. More broadly, this study enhances our understanding of how aging affects the

vestibular cues used during stair negotiation and walking.

**Supplementary Information:**

The online version contains supplementary material available at 10.1007/s00221-025-07141-5.

## Introduction

Aging is often accompanied by declining sensory acuity that can have a negative effect on balance control, potentially increasing fall risk. How the central nervous system adapts or compensates for these changes is therefore important to understanding the etiology of aging-related falls. The vestibular system contributes to balance control by encoding motion and orientation of the head in space. By the age of 70 years, vestibular receptors can lose up to 40% of their hair cells (Richter 1980; Merchant et al. 2000) and the number of central and peripheral vestibular neurons can decrease significantly (Richter 1980; Alvarez et al. 2000). The functional consequences of such deterioration may contribute to the decreased coordination and the high rate of falls observed as we age (Burns et al. 2016). While intuitive, causally linking the two is challenging, partially due to the difficulty probing the vestibular contribution to movement control in contexts in which falls appear more likely, such as during stair negotiation, but also central compensation may limit the impact age-related peripheral vestibular deficits have on behavior.

Several behavioral and physiological measures of vestibular function, such as vestibulo-ocular reflex function (Wall et al. 1984; Stefansson and Imoto 1986; DiZio and Lackner 1990; Peterka et al. 1990; Paige 1991; Baloh et al. 1993; Furman and Redfern 2001; Li et al. 2015), cervical and ocular vestibular evoked myogenic potentials (Welgampola and Colebatch 2001; Ochi and Ohashi 2003; Zapala and Brey 2004; Su et al. 2004; Brantberg et al. 2007; Basta et al. 2008; Iwasaki et al. 2008; Tseng et al. 2010; Nguyen et al. 2010; Piker et al. 2011, 2013; Rosengren et al. 2011) and motion detection and discrimination thresholds (Seemungal et al. 2004; Roditi and Crane 2012; Peters et al. 2016; Bermúdez Rey et al. 2016), all exhibit varying degrees of age-related changes that could be attributed to central and/or peripheral vestibular deterioration. And, while such deterioration could have a significant behavioral impact, there is evidence to suggest that it may be lessened by adaptive compensatory mechanisms (Peterka et al. 1990; Welgampola and Colebatch 2002; Jahn et al. 2003; Dalton et al. 2014; Peters et al. 2016). For example, in monkeys with gentamicin-induced peripheral vestibular lesions, slow-phase velocity nystagmus is increased during electrical vestibular stimulation compared to the monkey’s pre-peripheral lesion behavior (Phillips et al. 2016). This increase in nystagmus has been proposed to result from increased central vestibular gain arising as an adaptive compensation to the peripheral impairment (Phillips et al. 2016). In humans, similar behavior may be observed in older adult’s muscle responses to electrical vestibular stimulation (Welgampola and Colebatch 2002; Jahn et al. 2003; Dalton et al. 2014). During standing, electric vestibular stimulation induces biphasic responses in individual muscles of the legs and horizontal forces acting at the feet (Britton et al. 1993; Fitzpatrick et al. 1994). The latter component of this biphasic muscle response tends to increase with age, which has led to the proposal that an increase in vestibular gain may act to compensate for age-related peripheral vestibular deterioration (Welgampola and Colebatch 2002; Dalton et al. 2014). Together, the parallels between these cross-species and cross-function data suggest that mechanisms central to the peripheral vestibular apparatus may act to compensate for peripheral vestibular impairment. However, in humans specifically, age related changes in vestibular gain may be frequency specific. For example, short-latency muscle responses (< 100ms) to galvanic vestibular stimulation (GVS) (Britton et al. 1993) decrease with age, exhibiting the opposite behavior as the latter medium-latency muscle responses (> 100 ms) (Welgampola and Colebatch 2002; Dalton et al. 2014). This paradoxical behavior could be explained by increased low-pass filtering of vestibular signals with age (Dalton et al. 2014), or as a compensatory selective increase in gain of low frequency vestibular signals (Dalton et al. 2014; Peters et al. 2016). Either way, such a change in the relative proportion of lower to higher frequency signal content has been observed to increase or maintain the size of the medium-latency component of the biphasic response while decreasing the size of the short-latency component of the biphasic response (Dakin et al. 2010, 2011).

Here our aim was two-fold: first, we sought to determine the efficacy with which we could quantify aging-related changes in vestibular influence during stair negotiation because (a) little is known regarding the vestibular contribution to balance control during stair negotiation, and (b) better understanding of the vestibular system’s role during stair negotiation could better inform interventions aimed toward reducing the frequency of falls on stairs. Mechanically, ascending and descending stairs can impose a greater challenge than over ground walking. Peak support moments during both stair ascent and descent exceed those observed during level walking (McFadyen and Winters 1988). For older adults, the knee-extensor moment required to lift or lower the body are closer to the individual’s maximal isometric capacity than younger adults (Reeves et al. 2008; Samuel et al. 2011) and in stair ascent these torques may exceed this capacity (Samuel et al. 2011). Such elevated demands likely reduce the consistency of the accelerations experienced at the head. Once the mean step acceleration pattern is removed, residual variance is lowest in level walking, higher during stair ascent, and highest during stair descent (MacNeilage and Glasauer 2017). This graded increase in residual variance may suggest a parallel rise in the reliance on vestibular cues as task difficulty grows.

Previously, we examined vestibular influence during treadmill locomotion (Blouin et al. 2011; Dakin et al. 2013) and here we use this technique descriptively to (a) identify when, during stair negotiation, vestibular cues modulate lower limb muscle activity, (b) determine how the vestibular contribution to muscle activation changes between tasks and with aging and finally (c) to compare vestibular modulation during stair negotiation to treadmill locomotion in order to describe how they might differ. To simplify description of changes in stimulus influence over frequency regions we define 0–10 Hz as the *lower band* and 10–25 Hz as the *upper band* of stimulation frequencies (Dakin et al. 2011). We hypothesized that in older adults, lower band vestibular signals would exert greater relative influence, and upper band signals lower relative influence, than in younger adults as measured by increased coherence between the electric vestibular stimulus and electromyographic muscle responses (Welgampola and Colebatch 2002; Dalton et al. 2014; Peters et al. 2016). Because our study could not rigorously test broad differences in coherence between conditions due to variation in residual head acceleration variance between conditions, we did not propose a formal hypothesis about these trends. We do, however, discuss our results in light of what might be predicted based on differences in residual variance between conditions but also highlight the design constraints in the discussion.

## Methods

### Participants

Thirty-two participants were recruited for this study from the university campus and neighboring community and fifteen young adults (9 female, 6 male, 24.0 ± 3.8 yrs, 1.69 ± 0.08 m, 66 ± 11 kg) and fifteen older adults (8 female, 7 male, 66.4 ± 4.9 yrs, 1.71 ± 0.09 m, 73 ± 11 kg) were included in the final analysis. One young adult was removed due to equipment failure and one older adult was unable to finish the experiment due to challenges associated with traversing the stairs. Participants had no known history of neurological injury or disease that would impair their ability to negotiate the stairs, and each provided informed, written consent prior to participation. All procedures conformed to the declaration of Helsinki and were approved by Utah State University’s Institutional Review Board (protocol #7952).

### Procedures

Upon arrival at the laboratory, participants were screened for physical capability using the Physical Activity Readiness Questionnaire (PAR-Q) and an Electrical Vestibular Stimulation Pre-Screening Questionnaire (Hannan et al. 2021a). Participants that met the requirements for participation were then fit with heel and toe switches, seven electromyographic recording electrodes, a pair of stimulating electrodes, and a harness.

Both young and older participants had a wireless force-sensitive resistor (Delsys Trigno, Natick, MA, USA) placed under the head of the first metatarsal of their left foot to demarcate foot contact with the stairs or treadmill. Following placement of the force-sensitive resistor, wireless electromyography (EMG) sensors (Delsys Trigno, Natick, MA, USA) were placed on participants’ left anterior tibialis, soleus, medial gastrocnemius, vastus medialis, rectus femoris, semimembranous, and gluteus medius muscles after the skin was shaved and cleaned with rubbing alcohol. These specific muscles were chosen based on our previous success recording vestibular influence during locomotion (Blouin et al. 2011; Dakin et al. 2013). Electrode locations were determined by first having the individual contract or perform a movement meant to induce activation of the muscle of interest. Then origin and insertion identified, and if necessary palpated, then the Trigno sensor (which contains both electrodes and a 10 mm inter electrode distance) placed on the center of the muscle belly with the electrodes parallel to the estimated fiber orientation. Both the force-sensitive resistor and EMG were collected using a sampling rate of 5000 Hz. Once the EMG sensors were placed, the left leg was wrapped with a thin layer of foam elastic pre-wrap to prevent electrode shake and general sensor dislodging during trials. Participants were then bilaterally fit with gel-coated 9 cm^2^ carbon-rubber electrodes (Covidien Uni-patch, Dublin, IE), over their mastoid processes to pass the electric stimulus to the mastoid processes and underlying vestibular nerve. Once the simulating electrodes were placed, the head and electrodes were wrapped using elastic foam pre-wrap to prevent electrode slippage during the experiment. We also placed two small stickers on the right side of each participant’s head to visually monitor participants’ head pitch during the experiment. The reason for this is that the direction of postural disturbance to electric vestibular stimulation (EVS) depends on participant’s head orientation relative to their body (Lund and Broberg 1983; Fitzpatrick and Day 2004; Cathers et al. 2005; Khosravi-Hashemi et al. 2019). One of the two stickers was placed above the ear 18° from Reid’s plane (the line from the eye to the external auditory meatus, (Fitzpatrick and Day 2004) and the other at the corner of the eye. Any time researchers observed the line intersecting the two stickers deviating from horizontal, they would instruct the participant to tilt their head nose up, or nose down, to bring this line back to horizontal. Participants were also instructed to face forward and to maintain the desired head pitch for the duration of the experiment to restrict the direction of perturbation from the stimulus to the frontal plane, primarily (Lund and Broberg 1983; Fitzpatrick and Day 2004; Cathers et al. 2005; Khosravi-Hashemi et al. 2019). Once the stimulating and recording electrodes were placed, participants were harnessed to a linear freely moving assistive track (Biodex FreeStep-Supported Ambulation System, Shirley, NY) located overhead. This system served as a safety mechanism to prevent falls during the experiment and its rope length could be adjusted in real time to follow the participants’ change in height as they traversed the stairs. During data collection, one of the researchers walked parallel to the participant, next to, but not on the stairs, to continuously adjust the safety ropes’ length as the participant traversed the stairs. A second researcher adjusted the position of the stimulating electrode’s cable to minimize the cable tension experienced by participants as they traversed the stairs. In the event of a fall, tension on the rope, due to the fall, would cause an assistive braking device to lock the supportive rope’s length, halting the fall before contact with the stairs or ground.

**Fig. 1:**
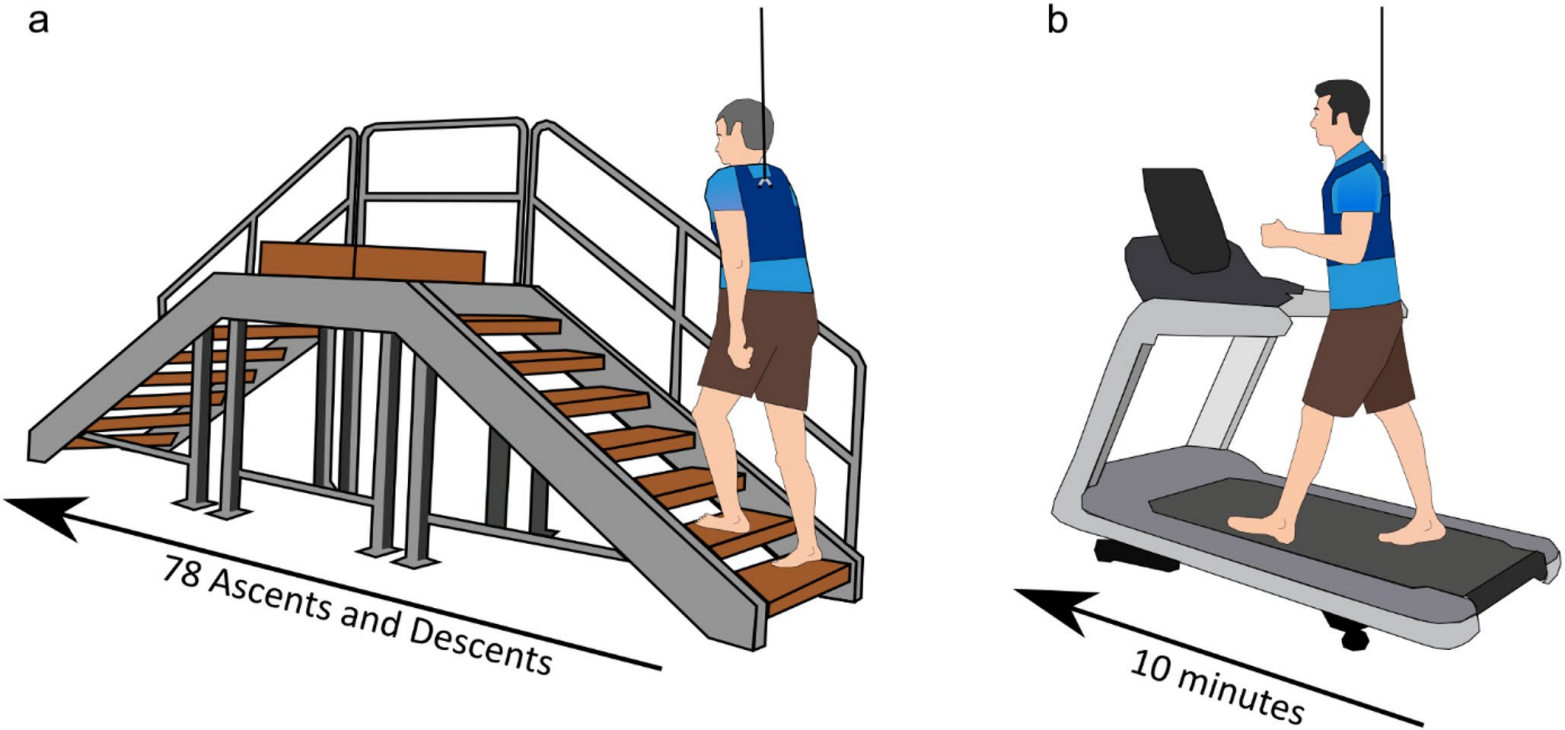
Tasks and methods for this study. **a** Participants ascended and descended a 9 - step staircase 78 times, with stimulation, to providing 312 steps, 300 of which were used for analysis. Participants wore a harness which was connected to a moving track above the stairwell. **b** Following stair negotiation, participants walked with stimulation on a treadmill for 10 min. During both stair negotiation and treadmill walking participants stepped at a metronome guided cadence of 76 steps/min

Prior to the beginning of the experiment, participants were acclimated to the stimulus through a series of three-second exposures to the stimulus. If the participant was uncomfortable with the stimulus, they could decline participation. Acclimation consisted of participant exposure to a 1-mA step-shaped waveform, a 2-mA step-shaped waveform, a 3-mA peak amplitude random waveform, a 4-mA peak amplitude random waveform, and a 5-mA peak amplitude random waveform. Once the acclimation was complete, participants traversed a nine-step staircase 78 times (Fig. 1a) with stimulation and then they completed 10-min walking on a treadmill (Fig. 1b) with stimulation. We chose to have participants complete the stair negotiation task prior to treadmill walking to ensure that older adults were not too fatigued to complete the stair negotiation task by the end of the experiment. This decision has the consequence that adaptation/habituation to the stimulus (Balter et al. 2004a, b; Kim 2009; Hannan et al. 2021a), or fatigue related changes in the stimulus response relationship weren’t distributed evenly across conditions, the impact of such being most prevalent during treadmill walking. During trials, participants received a 5 mA (peak-to-peak amplitude), bandwidth-limited random waveform stimulus (0–25 Hz, (Dakin et al. 2007) in a binaural bipolar electrode format. The stimulus was generated using Labview software (Bitter et al. 2006) by low-pass filtering white noise signal using a dual-pass 4th order Butterworth filter, with a cutoff of 25 Hz. The signal was then rescaled to have a peak-to-peak amplitude of 5 mA. To maintain continuity with our earlier locomotion studies (Blouin et al. 2011; Dakin et al. 2013), we used a 0–25 Hz stimulus bandwidth; the Discussion’s Future Research section explains why a 2–25 Hz range may be preferable going forward.

### Stairs

Each ascent and descent of the staircase provided 4 strides; a stride being left toe contact to left toe contact (Fig. 1a). We used 300 strides in the analysis, with the extra 12 strides available to substitute into the analysis to replace a stumble or mis-step. The staircase’s dimensions were within the international residential code (6.75-inch height and 12-inch depth, Fig. 1) (Anon 2015) and it had a handrail along the length of participants’ right side (Fig. 1a). Each participant ascended and descended the staircase one step at a time, placing one foot on each step and paced by a metronome to maintain an average cadence of 76 steps/min (Blouin et al. 2011; Dakin et al. 2013). At the end of stair ascent and the start of stair descent, participants were required to pause for two beats of the metronome to distinguish between the end of the ascent and the start of descent in post-processing. Note that while on the top landing, participants were allowed a brief downward glance to visually align themselves with the steps before beginning their descent. Following each pass over the staircase, participants were permitted a break if requested, and in the event of unsteadiness, participants were permitted to grasp the handrail to their right side. Trials in which the handrail was grasped were removed from the analysis. Each pass over the stairs took approximately 20 s and therefore the stimulus was delivered for 22s, the extra 2s to account for cadence errors. The stimulus was not provided between passes over the stairs. Researchers instructed participants to start stair ascent once the stimulus began and participants always started ascent and descent with their leg.

### Treadmill

Following the completion of the stair negotiation trials, participants walked on a treadmill (Fig. 1b) for an additional 10-min at a metronome guided cadence of 76 steps/min and a velocity of 0.4 m/s. Participants walked on a treadmill rather than over ground to reduce the duration of the experiment in order to limit fatigue in the older adults. We chose this velocity and cadence based on its prior successful use examining vestibular influence on muscle activity during locomotion (Blouin et al. 2011; Dakin et al. 2013). The stimulus was delivered over the entire 10-min walking period. However, only 300 strides were analyzed to be comparable with the number of steps collected during stair ascent and descent.

### Data analysis

We quantified the influence of the vestibular stimulus on lower limb muscle activity over the stride cycle using time-dependent measures of coherence and gain assuming a linear relationship between the stimulus and response (Forbes et al. 2014; Hannan et al. 2021b). First EMG from each condition in each subject were divided by its root mean square to normalize for EMG amplitude differences between participants. Data in each task were then cut into strides synchronized to the left toe strike using the wireless force-sensitive resistor placed on the foot. Toe contact was identified visually using a custom MATLAB script. Each segment was padded with an additional 25% of the preceding and subsequent stride to reduce distortion in the coherence and gain measures at the start and end of each stride. The data were then high-pass filtered at 30 Hz, rectified and then down-sampled to 200 Hz. Each muscles’ rectified EMG signal (Dakin et al. 2014), and the stimulus signal, during each step were converted to the frequency domain using a Morlet wavelet transform using a modified version (Blouin et al. 2011) of the methods outlined by (Zhan et al. 2006). To account for stride-to-stride variability, stride duration was normalized in time by resampling the cross and auto-spectra to the average stride length (Blouin et al. 2011; Dakin et al. 2013). Time-normalized time-dependent coherence was then averaged over steps within each subject to provide a single time-frequency estimate of coherence and gain for each subject.

Prior to comparing groups or conditions, the time-dependent coherence values were compared to zero using a cluster-based permutation test to identify regions of coherence that were significantly different from zero. Regions of significant time-frequency coherence were used to mask differences between conditions in order to highlight regions where a significant relationship (significant coherence) between the stimulus and muscles’ activity exists for at least one of the two conditions or groups being compared. To perform the permutation test we first calculated the empirical mean coherence across subjects within each condition. The data were permuted by independently shuffling the time and frequency coordinates of the coherence values for each subject and then calculating the mean across subjects of the shuffled data (the permuted means). This was repeated 10,000 times. From the permuted means the 95% percentile was determined and used as a threshold to identify time-frequency values in the empirical mean that exceed this threshold. The 95th percentile was chosen over the 99th percentile to expand the time-frequency space examined for significant differences in the cluster-based permutation tests comparing conditions and groups. The thresholded empirical data (a binary array) were clustered using MATLAB’s DBSCAN algorithm, with an epsilon value of 5 and a minimum cluster size of 25 points. DBSCAN groups nearby points based on density, identifying dense regions as clusters while labeling isolated points as noise. It automatically determines the number of clusters and can detect irregularly shaped groups without requiring prior knowledge of how many exist. The coherence values within each identified cluster were then summed to get a test statistic for each cluster that estimates the strength of the effect (stimulus - muscle relationship) over each cluster. Then each permuted mean was thresholded similarly to the empirical mean, clustered using similar DBSCAN parameters to the empirical mean and the sum of coherence within the permuted clusters was determined. The largest cluster from each permutation was retained. From the vector of largest clusters, the 95% percentile was estimated and used as the significant cluster threshold. Empirical clusters with coherence sums exceeding the threshold were defined as significantly different from zero. The coordinates for regions of non-significant coherence were then used as masks to highlight coherence and gain comparisons at coordinates exhibiting coherence significantly different from zero in at least one of the conditions. This is particularly important for the measure of gain as it is only meaningful when there is a relationship between the two signals.

Time-dependent coherence and gain were compared between age groups and conditions (stair ascent, stair descent, and walking) using a similar cluster-based permutation test (Maris and Oostenveld 2007). First, if necessary, the longer of the two time-frequency estimates were down sampled along the time axis to ensure data being compared were of similar dimensions in time and frequency. The significant coherence masks (described in the previous paragraph) were similarly down sampled to maintain their temporal relationship to the data they’re masking and were used in the figures illustrating the group or condition comparisons to highlight regions where a ‘coherent’ relationship exists. To identify clusters associated with potential significant differences between groups, data were thresholded by comparing the measure (coherence or gain), at each time frequency coordinate, between the two conditions or groups using a non-parametric statistical test. When comparisons were made between age groups, the data were thresholded based on the results of a two-tailed Wilcoxon rank sum test (*p* < 0.05), when data were compared within groups but between conditions, they were thresholded based on the results of a two-tailed Wilcoxon signed rank test (*p* < 0.05). After the non-parametric test, the test statistic array was thresholded at each time-frequency coordinate based on the test’s critical value for *p* < 0.05, creating a binary array that indicates whether the measure for a specific coordinate surpasses the significance threshold. This binary significance mask was then clustered using MATLAB’s DBSCAN clustering algorithm using the same parameters as the coherence significance test reported above (an epsilon of 5 and the minimum number of points in a cluster set to 25). The permutation test used to compare groups was distinct from the method employed to determine whether coherence was significantly different from zero. For group comparisons, participant group IDs were randomly permuted, and the statistical tests described above were used to compare the permuted groups, and the test type depending on the comparison made. This procedure aligns with the null hypothesis that any observed group differences are due to random assignment rather than systematic effects of the experimental condition. The resulting test statistics were thresholded using the statistical test’s critical value (*p* < 0.05) to create a binary threshold map for the permuted group comparisons, which was clustered in the same manner as the empirical data. For each permutation, the absolute values of the test statistics within each identified cluster were summed, and the largest cluster sum was retained. After completing all permutations, the empirical cluster sums were compared to the distribution of the largest cluster sums from the permutations (the largest cluster from each permutation). The p-value for each empirical cluster was then determined as its percentile rank within the sorted distribution of permuted cluster sums.

Lastly, electromyographic traces from each muscle were also down-sampled to a common length, rectified, normalized to the max value of the mean in each participant for each muscle, and in each condition, and presented with bootstrapped 95% confidence intervals using the percentile method (Efron and Tibshirani 1994).

## Results

One of the concerns with completing this experiment was whether older adults would be able to complete all 78 ascents and descents of the staircase. Indeed, one participant was unable to complete the experiment; however, the remainder did not explicitly indicate that the task was overly difficult. If a participant required a break, they were permitted to sit until ready to continue. In addition, while the stimulus is a mild disturbance to posture, it did not appear to challenge participants’ ability to complete the stair negotiation task. In fact, while there were more stumbles in the older adults (visually assessed as a misstep during collection), the average number was fairly small: 3.2 ± 2.0 stumbles or missteps per 312 total steps versus 0.5 ± 0.8 stumbles or missteps per 312 total steps in younger adults. There were no falls during the experiment.

When interpreting coherence results, it’s important to note that coherence reflects the proportion of variance in muscle activity linearly related to the stimulus. A decrease in coherence could result either from reduced vestibular input to the muscle or from increased non-vestibular contributions. Additionally, when comparing gains across conditions, variations in overall motor neuron excitability might influence gain amplitude, independent of vestibular function. Lastly, both coherence and gain measures primarily capture linear associations and may not effectively reflect nonlinear stimulus relationships. We therefore approach the following comparisons with caution, taking these limitations into consideration. In addition, throughout the results we will present the cluster means ($\:\stackrel{-}{x}$) and interquartile ranges (IQR) for the largest clusters in the upper and lower bandwidth when particular muscles are described; however, not all clusters will be presented in the text. Those that are not presented can be seen in supplementary Figs. 1, 2, 3, 4.

### Between age group comparisons

#### Treadmill

**Fig. 2:**
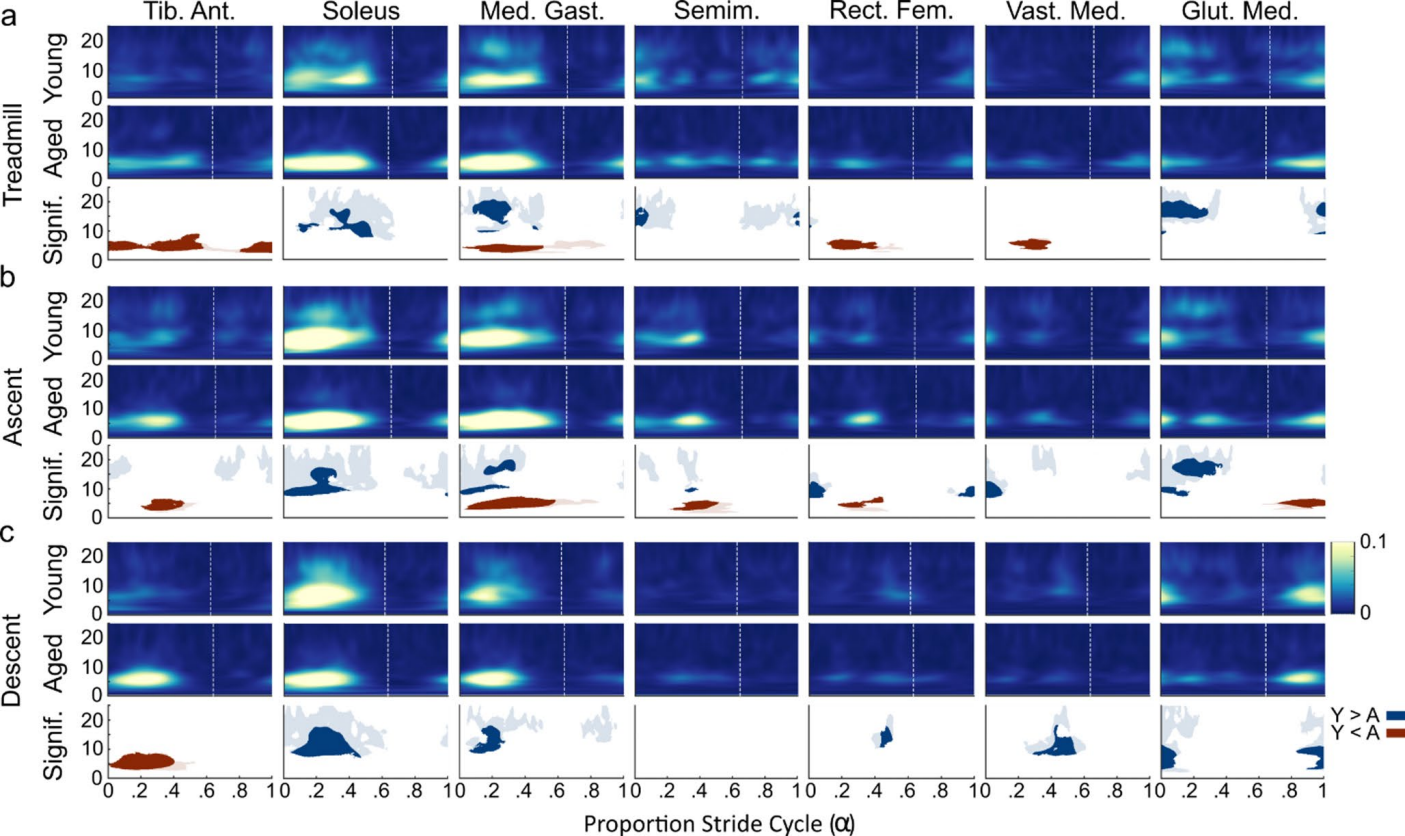
Time-frequency coherence compared between age groups. **a**. Treadmill: *Row 1*: Time-frequency coherence for young adults; *Row 2*: Time-frequency coherence for older (Aged) adults; *Row 3*: Coherence comparison using a cluster-based permutation test between age groups. A, B and C’s rows are organized similarly. Statistically greater coherence in young adults is in blue and in older adults is in red. Coherence was greater between 0–10 Hz in older adults in the tibialis anterior, soleus, medial gastrocnemius, rectus femoris and vastus medialis. Whereas coherence was greater from 10–25 Hz in young adults in the soleus, medial gastrocnemius and gluteus medius. **b**. Stair ascent. Coherence was greater between 0–10 Hz in older adults in the tibialis anterior, medial gastrocnemius, semimembranosus and gluteus medius. Coherence was greater from 10–25 Hz in young adults in the soleus, medial gastrocnemius and gluteus medius. **c**. Stair descent. Coherence was greater between 0–10 Hz in older adults in the tibialis. Coherence was greater from 10–25 Hz in young adults in the soleus, medial gastrocnemius, rectus femoris and vastus medialis. Also, coherence was greatest in young adults in the 0–10 Hz bandwidth in the gluteus medius. The x-axis represents the proportion of the step cycle, beginning and ending at toe contact. The vertical white dotted line marks the average timing of left toe-off within the step cycle (Young Adults– Treadmill: 66%, Stair Ascent: 64%, Stair Descent: 63%; Older Adults– Treadmill: 64%, Stair Ascent: 65%, Stair Descent: 64%). Med. Gast. - Medial Gastrocnemius; Tib. Ant. - Tibialis Anterior; Semim. - Semimembranosus; Rect. Fem. - Rectus Femoris; Vast. Med. - Vastus Medialis; Glut. Med. - Gluteus Medius. Young– Young adults; Aged - Older adults

**Fig. 3:**
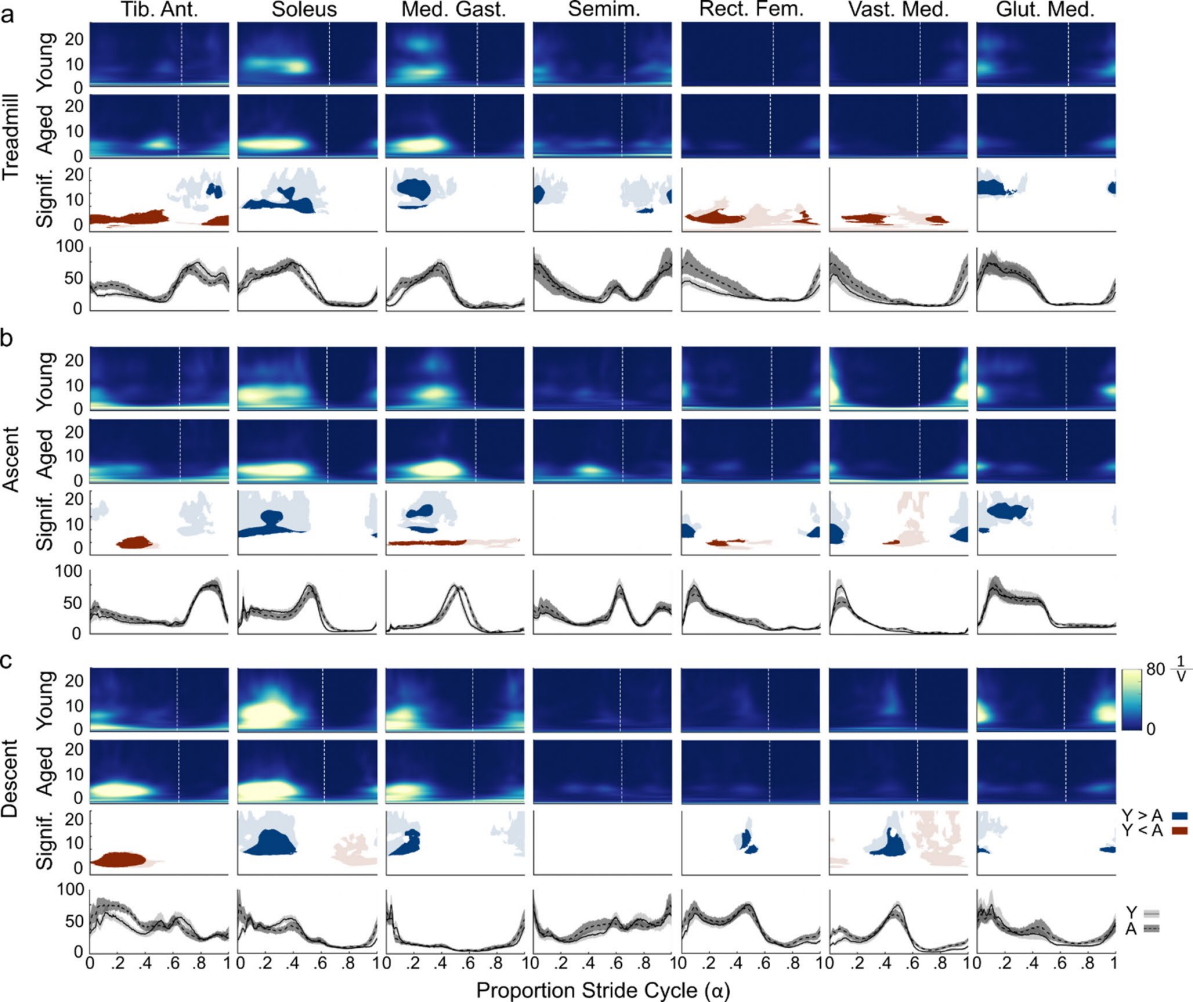
Time-frequency gain compared between age groups with normalized EMG plots. **a**. Treadmill: *Row 1*: Time-frequency gain plots for younger adults; *Row 2*: time-frequency gain plots for older (Aged) adults; *Row 3*: Comparison of gain between age groups using a cluster-based permutation test. A, B and C’s rows are organized similarly. Statistically greater gain in younger adults is in blue and in older adults is in red. *Row 4*: Electromyographic (EMG) activity over the step cycle for the two groups and bootstrapped 95% confidence interval. Younger adults are displayed using solid line with a light grey confidence interval and older adults have displayed using a segmented line and dark grey confidence interval. Gain was greater between 0–10 Hz in older adults in the tibialis anterior, soleus, rectus femoris and vastus medialis. Gain was greater from 10–25 Hz in young adults in the medial gastrocnemius and gluteus medius. **b**. Stair ascent. Gain was greater between 0–10 Hz in older adults in the tibialis anterior, medial gastrocnemius and cautiously in rectus femoris and vastus medialis. Gain was greater from 10–25 Hz in young adults in the soleus, medial gastrocnemius and gluteus medius. **c**. Stair descent. Gain was greater between 0–10 Hz in older adults in the tibialis anterior and. Gain was greater from 10–25 Hz in young adults in the soleus and vastus medialis. The x-axis represents the proportion of the step cycle, beginning and ending at toe contact. The vertical white line indicates toe-off. Med. Gast. - Medial Gastrocnemius; Tib. Ant. - Tibialis Anterior; Semim. - Semimembranosus; Rect. Fem. - Rectus Femoris; Vast. Med. - Vastus Medialis; Glut. Med. - Gluteus Medius. Y or Young– Young adults; A or Aged - Older adults

In general, the temporal pattern of GVS-muscle coherence between young and older adults was very similar but there was also a general trend of older adults having greater coherence (Red: Fig. 2a) in the lower band (0–10 Hz) than young adults whereas younger adults exhibited greater coherence (Blue: Fig. 2a) in the upper band (10–25 Hz) than older adults. Specifically, clusters of significantly increased lower band coherence were observed in the tibialis anterior (Aged: $\:\stackrel{-}{x}$ = 0.039, IQR = 0.015; Young: $\:\stackrel{-}{x}$ = 0.014, IQR = 0.009), medial gastrocnemius (Aged: $\:\stackrel{-}{x}$ = 0.058, IQR = 0.039; Young: $\:\stackrel{-}{x}$ = 0.029, IQR = 0.018), rectus femoris (Aged: $\:\stackrel{-}{x}$ = 0.03, IQR = 0.019; Young: $\:\stackrel{-}{x}$ = 0.008, IQR = 0.009) and vastus medialis (Aged: $\:\stackrel{-}{x}$ = 0.025, IQR = 0.026; Young: $\:\stackrel{-}{x}$ = 0.007, IQR = 0.006) of older adults. Younger adults exhibited significantly greater upper band coherence, compared to older adults, in the left soleus (Aged: $\:\stackrel{-}{x}$ = 0.007, IQR = 0.003; Young: $\:\stackrel{-}{x}$ = 0.023, IQR = 0.026), medial gastrocnemius (Aged: $\:\stackrel{-}{x}$ = 0.009, IQR = 0.005; Young: $\:\stackrel{-}{x}$ = 0.027, IQR = 0.022), and gluteus medius (Aged: $\:\stackrel{-}{x}$ = 0.005, IQR = 0.003; Young: $\:\stackrel{-}{x}$ = 0.026, IQR = 0.023). Lower band gain was also significantly greater in older adult’s left tibialis anterior (Aged: $\:\stackrel{-}{x}$ = 27.0, IQR = 13.3; Young: $\:\stackrel{-}{x}$ = 6.6, IQR = 6.1), rectus femoris (Aged: $\:\stackrel{-}{x}$ = 4.1, IQR = 1.6; Young: $\:\stackrel{-}{x}$ = 0.6, IQR = 0.3) and vastus medialis (Aged: $\:\stackrel{-}{x}$ = 2.8, IQR = 2.5; Young: $\:\stackrel{-}{x}$ = 1.1, IQR = 0.9) compared to younger adults (Red: Fig. 3a). The differences in upper band gain were similar to the differences in coherence, being greater in younger adult’s soleus (Aged: $\:\stackrel{-}{x}$ = 6.5, IQR = 6.9; Young: $\:\stackrel{-}{x}$ = 27.2, IQR = 31.9), medial gastrocnemius (Aged: $\:\stackrel{-}{x}$ = 4.0, IQR = 3.1; Young: $\:\stackrel{-}{x}$ = 22.2, IQR = 27.5), semimembranosus (Aged: $\:\stackrel{-}{x}$ = 1.4, IQR = 2.3; Young: $\:\stackrel{-}{x}$ = 11.7, IQR = 8.8) and gluteus medius (Aged: $\:\stackrel{-}{x}$ = 0.6, IQR = 1.4; Young: $\:\stackrel{-}{x}$ = 14.2, IQR = 5.2) during left leg stance (Blue: Fig. 3a). Note that weight bearing stance in the left leg lasted until approximately 66% of the stride cycle in younger adults and 64% in older adults and can be identified by the vertical white line on Figs. 2 and 3. Also, see the supplementary figures for cluster’s mean coherence and gain.

#### Stair ascent

Much like treadmill walking, older adults tended again to have greater lower band GVS-muscle coherence (Red: Fig. 2b) than younger adults and younger adults exhibited greater upper band GVS-muscle coherence (Blue: Fig. 2b) than older adults. Similar to walking, coherence across most muscles was highest when the left foot was in contact with the ground (Fig. 2b). Gluteus medius, however, tended to exhibit the highest coherence just before and during left foot contact with the step. Stimulus-muscle gain was greatest in the left soleus, medial gastrocnemius and tibialis anterior with older adults exhibiting higher lower band gain than younger adults (Red: Fig. 3b). Similar to coherence, the greatest gain was observed during periods when the left foot was in contact with the step.

#### Stair descent

 During stair descent, coherence in the lower band was generally similar between the two groups, except in the tibialis anterior. Older adults exhibited a prominent region of higher tibialis anterior coherence during stair descent left leg support that was significantly larger than in young adults (Red: Fig. 2c; Aged: $\:\stackrel{-}{x}$ = 0.07, IQR = 0.06; Young: $\:\stackrel{-}{x}$ = 0.02, IQR = 0.02). In contrast, younger adults exhibited greater upper band coherence in the left soleus (Aged: $\:\stackrel{-}{x}$ = 0.012, IQR = 0.01; Young: $\:\stackrel{-}{x}$ = 0.037, IQR = 0.032), medial gastrocnemius (Aged: $\:\stackrel{-}{x}$ = 0.009, IQR = 0.004; Young: $\:\stackrel{-}{x}$ = 0.025, IQR = 0.018), rectus femoris (Aged: $\:\stackrel{-}{x}$ = 0.003, IQR = 0.002; Young: $\:\stackrel{-}{x}$ = 0.012, IQR = 0.004) and vastus medialis (Aged: $\:\stackrel{-}{x}$ = 0.004, IQR = 0.003; Young: $\:\stackrel{-}{x}$ = 0.014, IQR = 0.007) than older adults (Blue: Fig. 2c). Notably, in the left gluteus medius younger adults appear to have greater low-band stimulus-muscle coherence than older adults around left toe contact (Aged: $\:\stackrel{-}{x}$ = 0.013, IQR = 0.01; Young: $\:\stackrel{-}{x}$ = 0.036, IQR = 0.028). Differences in gain between the two groups was largely similar to those observed in coherence, with the most prominent difference being that older adults exhibited greater lower band gain in the left tibialis anterior (Aged: $\:\stackrel{-}{x}$ = 83.6, IQR = 69.4; Young: $\:\stackrel{-}{x}$ = 17.3, IQR = 12.4) than young adults and younger adults exhibiting prominent greater upper band gain than older adults in the soleus (Aged: $\:\stackrel{-}{x}$ = 19.6, IQR = 20.5; Young: $\:\stackrel{-}{x}$ = 66.5, IQR = 56.5), medial gastrocnemius (Aged: $\:\stackrel{-}{x}$ = 6.7, IQR = 3.2; Young: $\:\stackrel{-}{x}$ = 24.7, IQR = 31.8), and vastus medialis (Red: Fig. 3c; Aged: $\:\stackrel{-}{x}$ = 2.2, IQR = 1.7; Young: $\:\stackrel{-}{x}$ = 14.2, IQR = 15.4).

### Within age group between condition comparisons: coherence

#### Young adults

**Fig. 4:**
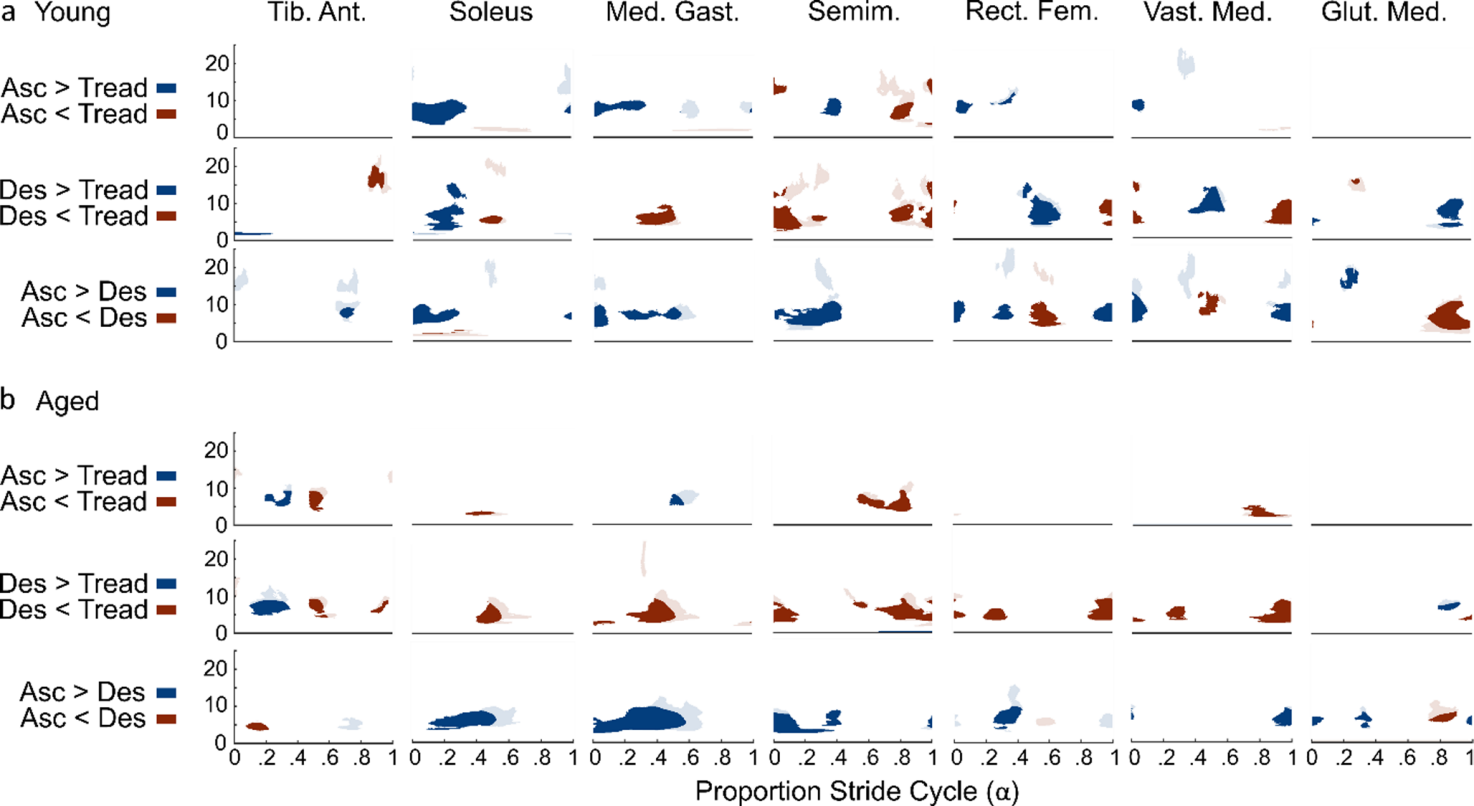
Statistical difference plots comparing coherence between conditions based on the cluster-based permutation test. Statistically different regions of coherence during periods where one or both conditions have statistically significant coherence are presented using saturated colors whereas differences outside regions of significant coherence are presented using less saturated colors. **a**. Coherence comparison between conditions in young adults. *Row 1* compares stair ascent to treadmill walking with statistically greater coherence during ascent in blue and greater coherence during treadmill walking in red. *Row 2* compares stair descent to treadmill walking with statistically greater coherence during descent in blue and greater coherence during treadmill walking in red. *Row 3* compares stair ascent to stair descent with statistically greater coherence during ascent in blue and greater coherence during stair descent in red. **b**. coherence comparison between conditions in older adults. *Row 1* compares stair ascent to treadmill walking with statistically greater coherence during ascent in blue and greater coherence during treadmill walking in red. *Row 2* compares stair descent to treadmill walking with statistically greater coherence during descent in blue and greater coherence during treadmill walking in red. *Row 3* compares stair ascent to stair descent with statistically greater coherence during ascent in blue and greater coherence during stair descent in red. The x-axis represents the proportion of the step cycle, beginning and ending at toe contact. Toe-off occurs at approximately ~65% (0.65) of the cycle. Asc. - Stair Ascent; Des. - Stair Descent; Tread. - Treadmill Walking; Med. Gast. - Medial Gastrocnemius; Tib. Ant. - Tibialis Anterior; Semim. - Semimembranosus; Rect. Fem. - Rectus Femoris; Vast. Med. - Vastus Medialis; Glut. Med. - Gluteus Medius

Young adults did not exhibit consistent changes in coherence across muscles between conditions. *Stair Ascent versus Treadmill* (Fig. 4a Row 1): Generally, coherence was greater during stair ascent than treadmill walking (Blue), except just prior to left toe contact, where coherence in the left semimembranosus (Asc: $\:\stackrel{-}{x}$ = 0.007, IQR = 0.005; Tread: $\:\stackrel{-}{x}$ = 0.023, IQR = 0.02) was greater during treadmill walking than stair ascent (Red). *Stair Descent versus Treadmill* (Fig. 4a Row 2): Coherence was greater in left soleus (Desc: $\:\stackrel{-}{x}$ = 0.116, IQR = 0.074; Tread: $\:\stackrel{-}{x}$ = 0.061, IQR = 0.044), rectus femoris (Desc: $\:\stackrel{-}{x}$ = 0.019, IQR = 0.019; Tread: $\:\stackrel{-}{x}$ = 0.005, IQR = 0.003), and vastus medialis (Desc: $\:\stackrel{-}{x}$ = 0.02, IQR = 0.025; Tread: $\:\stackrel{-}{x}$ = 0.005, IQR = 0.002) at left leg support and near toe-off during stair descent compared to treadmill walking (Blue), whereas coherence was greater in the semimembranosus (Desc: $\:\stackrel{-}{x}$ = 0.008, IQR = 0.009; Tread: $\:\stackrel{-}{x}$ = 0.039, IQR = 0.034) during left leg support and just prior to toe contact, and in medial gastrocnemius (Desc: $\:\stackrel{-}{x}$ = 0.025, IQR = 0.016; Tread: $\:\stackrel{-}{x}$ = 0.068, IQR = 0.053) just before toe-off during treadmill walking compared to stair descent (Red). *Stair Ascent versus Descent* (Fig. 4a Row 3): Early and mid-stance coherence in the left leg was generally greater during stair ascent than during stair descent (Blue) whereas coherence was greater in left rectus femoris (Asc: $\:\stackrel{-}{x}$ = 0.006, IQR = 0.004; Desc: $\:\stackrel{-}{x}$ = 0.022, IQR = 0.024) and vastus medialis (Asc: $\:\stackrel{-}{x}$ = 0.007, IQR = 0.008; Desc: $\:\stackrel{-}{x}$ = 0.021, IQR = 0.025) in late left leg support during stair descent compared to ascent (Red). Similarly, coherence was greater just before left foot contact in the left gluteus medius (Asc: $\:\stackrel{-}{x}$ = 0.019, IQR = 0.011; Desc: $\:\stackrel{-}{x}$ = 0.052, IQR = 0.035) during stair descent versus ascent.

#### Older (aged) adults

Older adults generally exhibited more consistent differences in coherence between conditions than younger adults. *Stair Ascent versus Treadmill* (Fig. 4b Row 1): Stair ascent and treadmill walking had the most similar coherence profiles across comparisons; the notable differences were regions of significantly higher coherence during treadmill walking near the end of the stride in semimembranosus (Asc: $\:\stackrel{-}{x}$ = 0.006, IQR = 0.003; Tread: $\:\stackrel{-}{x}$ = 0.026, IQR = 0.018) and vastus medialis (Asc: $\:\stackrel{-}{x}$ = 0.006, IQR = 0.004; Tread: $\:\stackrel{-}{x}$ = 0.017, IQR = 0.015) (Red) and in stair ascent mid stride in medial gastrocnemius (Asc: $\:\stackrel{-}{x}$ = 0.042, IQR = 0.04; Tread: $\:\stackrel{-}{x}$ = 0.015, IQR = 0.014) (Blue). *Stair Descent versus Treadmill* (Fig. 4b Row 2): Coherence was generally greater during treadmill walking compared to stair descent where much of the differences occur around left toe contact and during left leg weight bearing stance. *Stair Ascent versus Descent* (Fig. 4b Row 3): In most muscles, coherence was greater during stair ascent compared to stair descent. Much of this greater coherence was just before and during left leg support, as well as near left toe-off.

### Within age group between condition comparisons: gain

#### Young adults

**Fig. 5:**
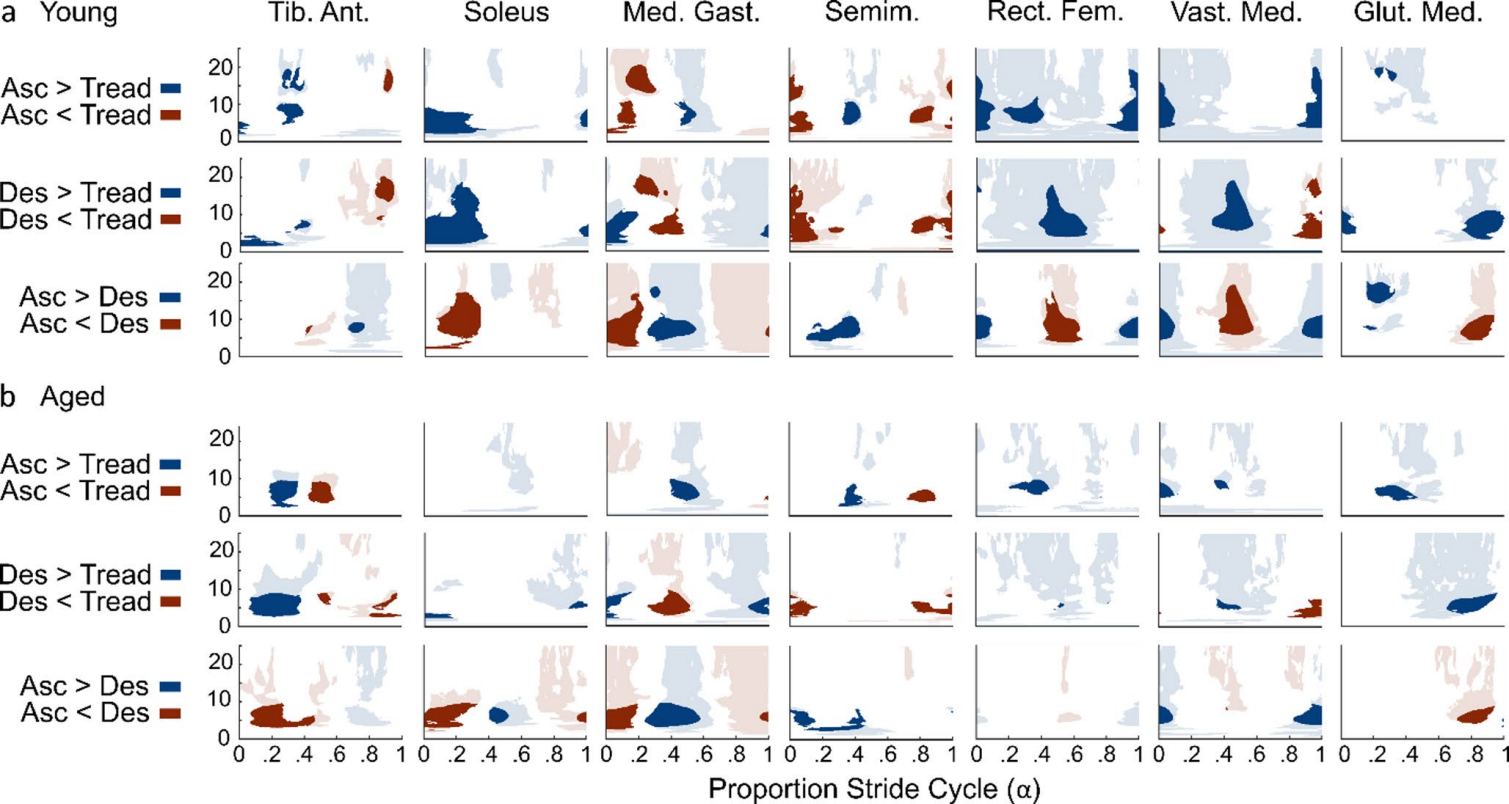
Statistical difference plots comparing gain between conditions based the cluster-based permutation test. Statistically different regions of gain during periods where one or both conditions have statistically significant coherence are presented using saturated colors whereas differences outside regions of significant coherence are presented using less saturated colors. **a** Gain comparison between conditions in young adults. Row 1 compares stair ascent to treadmill walking with statistically greater gain during ascent in blue and greater gain during treadmill walking in red. Row 2 compares stair descent to treadmill walking with statistically greater gain during descent in blue and greater gain during treadmill walking in red. Row 3 compares stair ascent to stair descent with statistically greater gain during ascent in blue and greater gain during stair descent in red. **b** Gain comparison between conditions in older adults. Row 1 compares stair ascent to treadmill walking with statistically greater gain during ascent in blue and greater gain during treadmill walking in red. Row 2 compares stair descent to treadmill walking with statistically greater gain during descent in blue and greater gain during treadmill walking in red. Row 3 compares stair ascent to stair descent with statistically greater gain during ascent in blue and greater gain during stair descent in red. The x-axis represents the proportion of the step cycle, beginning and ending at toe contact. Toe-off occurs at approximately ~65% (0.65) of the cycle. Asc. - Stair Ascent; Des. - Stair Descent; Tread. - Treadmill Walking; Tib. Ant. - Tibialis Anterior; Med. Gast. - Medial Gastrocnemius; Semim. - Semimembranosus; Rect. Fem. - Rectus Femoris; Vast. Med. - Vastus Medialis; Glut. Med. - Gluteus Medius

*Stair Ascent versus Treadmill* (Fig. 5a Row 1): Gain was generally greater during stair ascent versus treadmill walking and these differences were most prominent around left foot contact, with notable exceptions being in the medial gastrocnemius (Asc: $\:\stackrel{-}{x}$ = 8.0, IQR = 12.1; Tread: $\:\stackrel{-}{x}$ = 25.0, IQR = 30.6) and semimembranosus (Asc: $\:\stackrel{-}{x}$ = 5.6, IQR = 5.5; Tread: $\:\stackrel{-}{x}$ = 25.7, IQR = 31.5). *Stair Descent versus Treadmill* (Fig. 5a Row 2): Similar to stair ascent, descent also generally had greater gain in left leg muscles than treadmill walking with exception to the left medial gastrocnemius (Desc: $\:\stackrel{-}{x}$ = 16.7, IQR = 20.1; Tread: $\:\stackrel{-}{x}$ = 60.0, IQR = 52.8) and semimembranosus (Desc: $\:\stackrel{-}{x}$ = 2.02, IQR = 2.83; Tread: $\:\stackrel{-}{x}$ = 16.2, IQR = 16.5). *Stair Ascent versus Descent* (Fig. 5a Row 3): Differences in gain varied depending on the muscle with greater gain in descent during early left foot stance in soleus (Asc: $\:\stackrel{-}{x}$ = 37.0, IQR = 42.1; Desc: $\:\stackrel{-}{x}$ = 87.9, IQR = 61.9), medial gastrocnemius (Asc: $\:\stackrel{-}{x}$ = 8.9, IQR = 10.6; Desc: $\:\stackrel{-}{x}$ = 47.6, IQR = 47.8) and near toe off in rectus femoris (Asc: $\:\stackrel{-}{x}$ = 0.6, IQR = 0.5; Desc: $\:\stackrel{-}{x}$ = 5.5, IQR = 5.8) and vastus medialis (Asc: $\:\stackrel{-}{x}$ = 0.7, IQR = 0.7; Desc: $\:\stackrel{-}{x}$ = 12.5, IQR = 12.2) whereas medial gastrocnemius (Asc: $\:\stackrel{-}{x}$ = 44.4, IQR = 34.8; Desc: $\:\stackrel{-}{x}$ = 7.8, IQR = 8.7) and semimembranosus (Asc: $\:\stackrel{-}{x}$ = 9.0, IQR = 13.9; Desc: $\:\stackrel{-}{x}$ = 0.92, IQR = 0.5) exhibited greater gain in ascent in mid left leg stance, and in rectus femoris (Asc: $\:\stackrel{-}{x}$ = 16, IQR = 22; Desc: $\:\stackrel{-}{x}$ = 1.2, IQR = 1) and vastus medialis (Asc: $\:\stackrel{-}{x}$ = 74.0, IQR = 80.5; Desc: $\:\stackrel{-}{x}$ = 1.5, IQR = 1.6) near toe contact. Lastly, gluteus medius exhibited periods of greater gain during descent compared to ascent near left toe contact (Asc: $\:\stackrel{-}{x}$ = 6.7, IQR = 1.4; Desc: $\:\stackrel{-}{x}$ = 38.4, IQR = 18.8).

#### Older (aged) adults

*Stair Ascent versus Treadmill* (Fig. 5b Row 1): Older adults generally exhibited greater gain during stair ascent then treadmill walking where much of the significant difference was during left leg weight bearing stance. *Descent versus Treadmill* (Fig. 5b Row 2): Gain was greater during stair descent than treadmill walking in the left tibialis anterior (Desc: $\:\stackrel{-}{x}$ = 82.2, IQR = 67.5; Tread: $\:\stackrel{-}{x}$ = 13.6, IQR = 9.6) and gluteus medius (Desc: $\:\stackrel{-}{x}$ = 10.9, IQR = 11.9; Tread: $\:\stackrel{-}{x}$ = 2.5, IQR = 1.5) whereas gain was greater during treadmill walking in the left medial gastrocnemius (Desc: $\:\stackrel{-}{x}$ = 11.8, IQR = 12.0; Tread: $\:\stackrel{-}{x}$ = 57.3, IQR = 85.8) and semimembranosus (Desc: $\:\stackrel{-}{x}$ = 2.7, IQR = 2.2; Tread: $\:\stackrel{-}{x}$ = 18.6, IQR = 20.0). *Stair Ascent versus Descent* (Fig. 5b Row 3): Gain was greater in ascent than descent in the medial gastrocnemius (Asc: $\:\stackrel{-}{x}$ = 96.8, IQR = 103.5; Desc: $\:\stackrel{-}{x}$ = 12.0, IQR = 12.3) and vastus medialis (Asc: $\:\stackrel{-}{x}$ = 20.1, IQR = 16; Desc: $\:\stackrel{-}{x}$ = 1.7, IQR = 1.1) and greater in descent just after left toe contact in the left tibialis anterior (Asc: $\:\stackrel{-}{x}$ = 12.9, IQR = 19.9; Desc: $\:\stackrel{-}{x}$ = 82.2, IQR = 64.6), soleus (Asc: $\:\stackrel{-}{x}$ = 51.4, IQR = 78.7; Desc: $\:\stackrel{-}{x}$ = 116.8, IQR = 81.3) and medial gastrocnemius (Asc: $\:\stackrel{-}{x}$ = 19.0, IQR = 22.6; Desc: $\:\stackrel{-}{x}$ = 62.5, IQR = 43.7) and just prior to toe contact in gluteus medius (Asc: $\:\stackrel{-}{x}$ = 2.6, IQR = 2.6; Desc: $\:\stackrel{-}{x}$ = 15.1, IQR = 15.3).

## Discussion

Here we explored vestibular influence on muscle activity in the legs during stair negotiation and treadmill walking. We were generally successful at extracting vestibular influence during stair negotiation, providing a first look at vestibular influence on posture during stair negotiation in both younger and older adults. We found that compared to younger adults, older adults commonly exhibited greater GVS-muscle coherence at frequencies between 0 and 10 Hz but lower levels of coherence at frequencies between 10 and 25 Hz, supporting our hypothesis and prior observations of similar phenomena (Dalton et al. 2014). Between tasks (stair ascent, descent, and treadmill walking), older adults exhibited a more consistent pattern of changes in coherence than young adults. In the muscles recorded, coherence was generally greater during stair ascent than descent and during treadmill walking than during stair descent. There were, however, no consistent differences in pattern between stair ascent and treadmill walking in older adults. Overall, we demonstrated that, like during standing, aging changes the bandwidth with which vestibular signals influence balance control during stair negotiation and locomotion.

### Changes in the frequency bandwidth of vestibular-muscle coherence with age

One of the prominent differences we observed with aging was that the influence of stimulus frequencies between the 10–25 Hz declines with age while the influence of stimulus frequencies below 10 Hz increases with age. A shift in the relative proportion of lower band to upper band frequencies contributing to the response, as we observed here, could produce a larger medium-latency response and smaller short-latency response, as previously observed for older adults quiet standing compared to younger adults (Welgampola and Colebatch 2002; Dalton et al. 2014). Two non-mutually exclusive mechanisms have been proposed previously to underlie these changes. The first is that signals could undergo low-pass filtering resulting in reduced high-frequency signal content (Dalton et al. 2014). By itself, such a mechanism could result in reduced upper band stimulus-response coherence while sparing lower frequency stimulus-response coherence. Why this could happen is unclear, however one possibility is that there is a disproportionate reduction in the efficacy of the pathways transmitting transients or high frequency motion signals. Irregular vestibular afferents have significantly higher gain across stimulation frequencies than regular afferents (Kwan et al. 2019) which improves their encoding of natural stimuli (Schneider et al. 2015) and low gain irregular afferents appearing most sensitive to encoding the onset of rapid movement (Hullar et al. 2005). Irregular afferents have also been shown to be activated and phase lock to vibration (Curthoys et al. 2006; Curthoys 2017) highlighting their potential sensitivity to transient events (for topical review see: Curthoys et al. 2017). With aging, some studies have observed disproportionate type I hair cell loss in places like the central region of the cristae ampullaris, and in the maculae of the otoliths (Engstrom et al. 1974; Rosenhall 1973) where large irregular vestibular afferents are located (Zalewski 2015), suggesting that irregular afferents may be disproportionately affected. These findings raise a speculative but biologically plausible question: whether the age-related shift toward lower frequency coherence reflects a decrease in the transmission of higher-frequency information due to the disproportionate loss of irregular vestibular afferents. We note, however, that afferent’s responses to GVS differ across frequency from their responses to natural motion (Kwan et al. 2019) and therefore these modality-specific differences should be considered when evaluating the validity of these cross-modal comparisons.

A similar result could also be achieved by selectively boosting the gain of lower band signals (Dalton et al. 2014; Peters et al. 2016) in response to broad-bandwidth signal attenuation associated with age-related vestibular deterioration. Indeed, Peters et al. (2016) have proposed that frequencies from 1 to 5 Hz may be selectively boosted with age because the majority of natural motion signal power presides below 5 Hz (Grossman et al. 1988; Pozzo et al. 1990; Hirasaki et al. 1993; Carriot et al. 2017). Here we observed increases in low frequency coherence with age with a center frequency of approximately 5 Hz that appears congruent with the proposition of bandwidth specific boosting, but also might suggest that frequency-specific increases could extend beyond the 1–5 Hz proposed previously (Peters et al. 2016), possibly up to 10 Hz. However, it is unclear whether this ‘extension’ in bandwidth is limited to locomotor related activities.

Several researchers have proposed that changes in the bandwidth of vestibular - influence with aging may be centrally mediated (Peterka et al. 1990; Welgampola and Colebatch 2002; Jahn et al. 2003; Dalton et al. 2014; Phillips et al. 2016; Peters et al. 2016). Perhaps the strongest support for this premise arises from the increase in nystagmus observed in monkeys with gentamicin-induced peripheral vestibular lesions. Following the lesion, the sensitivity of the monkey’s vestibular afferents to electrical current was generally reduced, however slow phase velocity nystagmus caused by the electric stimulus was increased compared to the same monkey pre-lesion (Phillips et al. 2016). Because peripheral afferent sensitivity was decreased, the increased response gain could have been due to an increase in the sensitivity of secondary vestibular neurons to presynaptic input, resulting in their greater recruitment, though the authors indicate further empirical validation is necessary. In humans, however, gentamicin induced vestibular impairment is associated with a decrease in the amplitude of electrically evoked vestibulo-ocular reflexes (Aw et al. 2008). The attenuation of electrically evoked vestibulo-ocular reflexes in humans could suggest that central compensation did not occur or was insufficient to overcome gentamicin-related impairment. Regardless, these conflicting observations indicate that further investigation is required to establish the location and possible mechanisms associated with aging related vestibular adaptation.

Alternatively, because vestibular signals converge on the motor neuron pool, a common pathway to the muscle shared by visual, proprioceptive and other inputs, stimulus-correlated responses that appear at longer latencies are progressively more likely to be contributed to indirectly by the stimulus or from its behavioral consequences. Possible intermediaries include: postural responses to stimulus, postural responses to stimulus related eye movements or recurrent proprioceptive feedback generated by stimulus induced changes in muscle length and force. Such secondary loops could amplify low-frequency coherence in older adults even when the primary vestibular pathway is unchanged provided two conditions are met. First, the indirect response must remain phase locked to the stimulus at the same frequency (linearly related) and second the response must occur in the same 1-step window as the stimulus (left toe-strike to left toe-strike). Responses whose dynamics extend beyond this window will be attenuated in the coherence and gain estimates, which likely excludes most of the slower sway-related effects of the stimulus. Earlier work investigating off-axis (cross-frequency) correlations and other non-linear distortions in similar stochastic GVS paradigms found little evidence for strong non-linear coupling in horizontal ground reaction forces (Forbes et al. 2014; Hannan et al. 2021b) or medial gastrocnemius EMG (Forbes et al. 2014) during quiet standing. Whether the same behavior is observed during locomotor tasks is unclear.

Lastly, the age-related redistribution of GVS-EMG coherence toward lower frequencies may be partly explained by peripheral age-related adaptation in motor-unit and muscle properties rather than central changes in sensory weighting. With aging, motor unit numbers typically decline (Piasecki et al. 2016), firing rates decrease (Kirk et al. 2021; Rubinstein and Kamen 2005), neuromuscular junction transmission becomes more variable (Arnold and Clark 2023; Ham and Rüegg 2018), sarcolemma excitability decreases (Lee et al. 2018), skeletal muscle strength is reduced (Frontera et al. 2000), contraction velocity slows (Raj et al. 2010) and tendon stiffness decreases (Kwan et al. 2023) (For general review of aging related changes see: Wu et al. 2021). Collectively, these peripheral changes may amplify the low-pass filter characteristics of the periphery and decrease high-frequency information transmission due to increased variability. Specifically, variability in action potential timing potentially arising from aging-related changes such as motor neuron demyelination (Kim et al. 2013; Verdú et al. 2000), impaired neuromuscular junction function or reduced sarcolemma excitability, could disproportionately reduce coherence at higher frequencies (Neiman et al. 2011). Supporting this idea, Neiman et al., demonstrated, via simulation, that increased spike timing jitter disproportionately reduces coherence at higher frequencies. Thus, local jitter from peripheral adaptations could contribute to the observed age-related shift toward lower frequency GVS-EMG coherence. In contrast, varying motor unit firing rate and action potential shape were unable to explain differences in GVS-EMG coherence bandwidth between muscles in the neck, back, and lower limbs, suggesting these may not be dominant factors contributing to the shift in bandwidth observed with aging (Forbes et al. 2013). Further research is necessary to clarify the role of peripheral changes in the age-related shift in coherence bandwidth observed in this current study.

### Increased tibialis anterior role in balance control with age

One of our more novel observations is the increased GVS-muscle coherence observed in the left tibialis anterior of older adults during stair descent compared to younger adults. Large responses to GVS in the tibialis anterior have been observed previously in older adults during standing balance (Dalton et al. 2014). The heightened role of the tibialis anterior could represent a general change in the balance control strategy as we age, which in the particular case of stair descent, may also be associated with an effort to avoid falling forward during stair descent. Fear of falling is known to increase GVS-postural response coherence (Horslen et al. 2014; Lim et al. 2017), particularly when the postural disturbance caused by the stimulus increases the threat of a fall (Horslen et al. 2014). Here the stimulus creates a medio-lateral disturbance, which when directed to the left (there is no hand-rail on the left), could have increased perceived fall risk. The additional risk of falling forward down the stairs may have heightened this effect. Thus, larger GVS-tibialis anterior coherence may reflect increased incorporation of the tibialis anterior in balance control. Generally, the strength of the relationship between vestibular stimulus and response appears to increase when either head movement is less predictable (MacNeilage and Glasauer 2017) or when muscles are engaged in some form of feedback driven balance control (Britton et al. 1993; Fitzpatrick and Day 2004), therefore heightened involvement in balance control is a possible explanation. However, since the tibialis anterior acts to bring you forward, such a strategy could paradoxically heighten the risk of a forward fall. Alternatively, heightened vestibular coupling with the tibialis anterior may reflect an active increase in vestibularly derived joint stiffening around the ankle during descent to limit anterior posterior variability. Ultimately, more research is clearly needed to interpret the tibialis anterior’s specific contribution to stair descent.

### Consistent changes in vestibular involvement between conditions in older adults

Between conditions, older adults displayed relatively consistent changes in coherence across left leg muscles compared to younger adults. For example, coherence was generally greater during stair ascent than stair descent as well as during treadmill walking compared to stair descent whereas in younger adults, there was very little consistent change between conditions and the changes that did occur were specific to the muscle examined. Theoretically, overall vestibular influence on motor control during locomotor tasks has been proposed to rise as the stride-to-stride residual headmotion variance, the portion of the signal not captured by the mean stride pattern, increases and thus predictability decreases (MacNeilage and Glasauer 2017; Dietrich et al. 2020). Evidence from frogs and other vertebrates shows that predictive motor signals can replace or suppress unreliable sensory input. For example, in amphibians a copy of the locomotor command, termed an efference copy, a subtype of the broader corollarydischarge class (von Holst and Mittelstaedt 1950; Sperry 1950), can attenuate or substitute for vestibular feedback during stepping (Lambert et al. 2012; von Uckermann et al. 2013). Similar predictive mechanisms have been inferred in humans when sensory reliability falls (MacNeilage and Glasauer 2017). Because such gating could occur at spinal, brainstem, and cerebellar levels, it may not depend on cortically mediated higher level contextual information. Moreover, when stridetostride vestibular input is highly predictable, the CNS can favor these anticipatory signals, reducing the need for slower, feedbackdriven corrections. Thus, increased predictability is proposed to be associated with decreased vestibular influence. When applied to the results here, since residual head motion variability is reported to be greater during stair descent than during stair ascent (MacNeilage and Glasauer 2017), predictability should be lower during stair descent versus ascent, and thus GVS associated responses should be greater during descent compared to ascent. Similarly, the increased residual head variability reported during stair ascent compared to treadmill walking (MacNeilage and Glasauer 2017) suggests GVS associated responses should be greater during stair ascent compared to even-ground walking. While not much can be said about younger adults, because of the significant variability in the differences in coherence between muscles, in older adults, coherence was generally greater during stair ascent than descent and greater during treadmill walking than during stair descent. There did not appear to be any consistent differences in coherence between stair ascent and treadmill walking. At face value, these differences appear inconsistent with MacNeilage and Glasauer’s (2017) predictability-dependent theory of vestibular cue utilization (similarly noted in a recent walking study; Foulger et al. 2025), suggesting an alternative theory might have better explanatory power, however these discrepancies could also reflect limitations of the current study (described further below) rather than a definitive challenge to the theory or some mechanistic change associated with aging.

Step predictability may also depend on how much advance visual information each condition affords. With participants’ heads fixed 18° nose up, there was no foveal preview of the next foot placement, and the richness of peripheral cues varied by condition: stair ascent the richest, treadmill walking was at an intermediate level, and stair descent was the most impoverished. According to sensory reweighting (Asslander and Peterka 2014; Peterka 2002) theory, and an optimal cue integration framework, the nervous system down-weights unreliable sensory channels and up-weights the most precise channels (Ernst and Banks 2002; Fetsch et al. 2010). Therefore, one might expect visual up-weighting, and conversely, vestibular down-weighting, to be greatest during stair ascent. Our results do not appear to support this prediction, however. In younger adults, stimulus-muscle coherence showed no consistent pattern, making whole-body sensory weighting hard to infer. In older adults, coherence was higher for stair ascent than stair descent and for treadmill walking than stair descent, while gain peaked in stair ascent relative to both other tasks. These patterns conflict with a simple visual reliability based reweighting explanation. Alternatively, studies modelling postural control during standing have suggested that reliance on vestibular cues may be reduced in favor of proprioceptive cues in the elderly (Wiesmeier et al. 2015). Here older adults exhibited lower upper band coherence but higher lower band coherence suggesting either a frequency specific reweighting in older adults or potentially confounding effects in the later lower frequency influence of the stimulus (E.g., a down weighting of vestibular cues affecting the upper band frequencies but larger indirect stimulus related activity linearly influencing the muscle at lower band frequencies). Ultimately, it may also be that the task demands confound the resolution of possible re-weighting effects. Stair ascent imposes greater physical expenditure (McFadyen and Winter 1988; Teh and Aziz 2002) which could have altered vestibular or proprioceptive contributions relative to stair descent or other tasks like standing. Consequently, this experiment likely lacks the sensitivity to make strong statements about cross modal sensory weighting in its current format.

An alternative hypothesis for the differences between conditions is that predictable vestibular cues (re-afference) are essential for determining the current motor context specifically, whether vestibular information is actively used in the closed-loop control of the body, and therefore some degree of predictable vestibular cues are necessary for GVS to influence motor activity. This hypothesis arises from the observed association between a muscles’ involvement in balance or stability control and the presence of muscle responses to GVS (Britton et al. 1993; Fitzpatrick et al. 1994). A notable example arises from Luu et al. (2012) whom had participants balance on a 6-degree-of-freedom motion platform that behaved like an inverted pendulum. Participants controlled this platform by adjusting pressure with their feet on a force-plate, closely mimicking natural standing balance. When participants actively controlled the platform’s motion (i.e., vestibular feedback was coupled with their motor actions), significant coherence between GVS and soleus muscle activity emerged. However, when control was unknowingly switched to computer-generated movements (decoupling vestibular feedback from participants’ actions while participants seemingly believed they were still balancing the platform), the GVS-soleus coherence was significantly reduced. These findings suggest that predictable vestibular feedback may be required for unpredictable vestibular feedback to meaningfully influence motor behavior. One possible reason for this observation is that predictable vestibular information helps determine the current motor context (Heald et al. 2021)—for instance, whether the nervous system is in a state of feedback-driven control where vestibular signals are valuable to correct for errors in movement control or to compensate for unpredictable events. Thus, predictable vestibular signals may help the system infer that self-generated movement, like balancing, is occurring. In extreme cases this could lead to the gating of responses to GVS, like observed by Britton et al. (1993), Fitzpatrick et al. (1994) and Luu et al. (2012) but possibly also scaling the influence (gain) of vestibular feedback from unpredictable events (ex-afference), like GVS, to the demands of the task. Such a scaling of gain may help explain variance in the degree of GVS-muscle coupling observed across different muscles at any given point in time across the gait cycle as well as the association between GVS response size and the stabilization demands of a task (Magnani et al. 2021).

Alternatively, several limitations of this study may have contributed to the differences from MacNeilage and Glasauer (2017) that we observed. First, while we have recorded from several muscles, the sample of muscles that we have recorded may not accurately reflect the overall postural response (i.e. the total body response is the sum of responses from all muscles). Second, to increase safety, participants were harnessed to an overhead railing at all times. Changes in variance introduced by the supporting cable could have changed coherence amplitude, particularly if head variance decreased (MacNeilage and Glasauer 2017) or stability increased (Britton et al. 1993; Fitzpatrick et al. 1994). Alternatively, researcher error in adjusting the length of this supporting cable could have intermittently interfered with participants’ gait, introducing additional variability into the gait pattern. This was a necessary compromise to reduce the risk and consequences of a fall during stair negotiation. Third, the theoretical predictions of overall vestibular influence were made based on head movement during over-ground walking (MacNeilage and Glasauer 2017) and not during treadmill walking, as used here. Head motion predictability is likely lower during over ground walking (depending on the terrain) than during treadmill walking, which may alter the average magnitude of coherence and trends in coherence between conditions that we observed here.

### General limitations

Moreover, four additional limitations should be acknowledged. The first is that both participant groups performed the treadmill condition last to limit the fatigue experienced during stair negotiation. As it turned out, while there was likely some fatigue associated with the study, it did not appear to significantly contribute to participant attrition as no participants failed to complete the study due to fatigue. There are, however, two negative consequences associated with this decision. First participant fatigue would have been greatest during the treadmill walking which may have changed the spatiotemporal patterns of coherence during this condition. Second, prolonged exposure to the vestibular stimulus likely resulted in participant adaptation to the stimulus over time, which could have reduced overall coherence and gain amplitude during treadmill walking compared to stair ascent and descent (Balter et al. 2004b; Dilda et al. 2014; Hannan et al. 2021a) (Figs. 4 and 5). Because participant fatigue was not as big a confounding factor as anticipated, future studies should randomize the presentation of stair versus treadmill walking conditions. Third, participants were instructed to walk in time with the metronome, but were not instructed to walk with any particular gait pattern. If younger and older adults adopted different gait patterns (like older adults reaching for steps on descent), on average, this could alter the coherence and gain measures between the two. Lastly, electrode locations were determined manually in this study; adopting the standardized SENIAM proportional placement guidelines (Hermens et al. 2000) would likely have increased placement repeatability and may have improved signal quality.

### Future research

Given the success in extracting vestibular influence on muscle activity during stair negotiation, future work could investigate changes in vestibular influence on motor activity in older adults with a history of falling, with appropriate safety measures, to more directly determine if changes in vestibular influence could be associated with increased fall risk. Future research could also examine a broader distribution of muscles, or use instrumented stairs, in order to get a more wholistic view of vestibular influence on the body. Moreover, given our binaural bipolar electrode configuration (Fitzpatrick and Day 2004) selecting muscles with a larger role in medio-lateral stability, such as the peroneals (fibularis), might provide further insight into the whole-body vestibular compensation strategy. To clarify whether selective afferent loss underlies the coupling-bandwidth differences we observed, future work should combine vestibular-coupling measures with complementary functional probes (For review see Wagner et al. 2021), such as the video head-impulse test (Curthoys et al. 2023), psychophysical motion-detection thresholds (Kobel et al. 2021; Bermúdez-Rey et al. 2016), or targeted afferent-ablation animal models to better isolate the mechanistic source of these differences. Lastly, we used a 0–25 Hz bandwidth to match previous locomotor studies. The trade-off is that most EVS-induced sway occurs at frequencies below 2 Hz (Dakin et al. 2010), whereas much of the stimulus-muscle response coupling is concentrated above 2 Hz. Future work could therefore adopt a 2–25 Hz bandwidth: it would preserve the majority of stimulus-response information while minimizing low-frequency sway, improving participant safety, and likely reducing fatigue during long test sessions.

## Conclusion

We explored vestibular influence on muscle activity in the legs during stair negotiation and treadmill walking. We found that compared to younger adults, older adults generally exhibited greater vestibular stimulus - muscle coherence at frequencies between 0 and 10 Hz but lower levels of coherence at frequencies between 10 and 25 Hz. Between tasks older adults exhibited a more consistent pattern of changes in coherence between conditions compared to younger adults. Across muscles, coherence was greater during stair ascent than descent and coherence was greater during treadmill walking than during stair descent in the muscles recorded. Overall, these results demonstrate that vestibular influence on posture can be extracted during challenging tasks such as stair negotiation to better understand how these signals are used to coordinate movement and to examine changes that occur as we age.

## Supplementary Information

Below is the link to the electronic supplementary material.

Supplementary Material 1

Supplementary Material 2

Supplementary Material 3

Supplementary Material 4

## Data Availability

The data supporting the results of this study are available by reasonable request to the corresponding author.

## References

[CR1] Alvarez JC, Díaz C, Suárez C et al (2000) Aging and the human vestibular nuclei: morphometric analysis. Mech Ageing Dev 114:149–172. 10.1016/S0047-6374(00)00098-1 10802120 10.1016/s0047-6374(00)00098-1

[CR2] Anon (2015) 2015 international residential code, second. International Residential Code, USA

[CR3] Arnold WD, Clark BC (2023) Neuromuscular junction transmission failure in aging and sarcopenia: the nexus of the neurological and muscular systems. Ageing Res Rev 89:101966. 10.1016/j.arr.2023.101966 37270145 10.1016/j.arr.2023.101966 PMC10847753

[CR4] Asslander L, Peterka RJ (2014) Sensory reweighting dynamics in human postural control. J Neurophysiol 111:1852–1864. 10.1152/jn.00669.2013 24501263 10.1152/jn.00669.2013 PMC4044370

[CR5] Aw ST, Todd MJ, Aw GE et al (2008) Gentamicin vestibulotoxicity impairs human electrically evoked vestibulo-ocular reflex. Neurology 71:1776–1782. 10.1212/01.wnl.0000335971.43443.d9 19029517 10.1212/01.wnl.0000335971.43443.d9

[CR6] Baloh RW, Jacobson KM, Socotch TM (1993) The effect of aging on visual-vestibuloocular responses. Exp Brain Res 95:509–516. 10.1007/BF00227144 8224077 10.1007/BF00227144

[CR7] Balter SGT, Stokroos RJ, Akkermans E, Kingma H (2004a) Habituation to galvanic vestibular stimulation for analysis of postural control abilities in gymnasts. Neurosci Lett 366:71–75. 10.1016/j.neulet.2004.05.015 15265593 10.1016/j.neulet.2004.05.015

[CR8] Balter SGT, Stokroos RJ, Eterman RMA et al (2004b) Habituation to galvanic vestibular stimulation. Acta Otolaryngol (Stockh) 124:941–945. 10.1080/00016480410017350 15513531 10.1080/00016480410017350

[CR9] Basta D, Todt I, Ernst A (2008) Characterization of age-related changes in vestibular evoked myogenic potentials. J Vestib Res 17:93–98. 10.3233/VES-2007-172-304 18413902

[CR10] Bermúdez Rey MC, Clark TK, Wang W et al (2016) Vestibular perceptual thresholds increase above the age of 40. Front Neurol 7:1–17. 10.3389/fneur.2016.00162 27752252 10.3389/fneur.2016.00162 PMC5046616

[CR11] Bitter R, Mohiuddin T, Nawrocki M (2006) LabVIEW: advanced programming techniques. CRC Press, London

[CR12] Blouin J-S, Dakin CJ, Van Den Doel K et al (2011) Extracting phase-dependent human vestibular reflexes during locomotion using both time and frequency correlation approaches. J Appl Physiol 111:1484–1490. 10.1152/japplphysiol.00621.2011 21868684 10.1152/japplphysiol.00621.2011

[CR13] Brantberg K, Granath K, Schart N (2007) Age-related changes in vestibular evoked myogenic potentials. Audiol Neurotol 12:247–253. 10.1159/000101332 10.1159/000101332 17389791

[CR14] Britton TC, Day BL, Brown P et al (1993) Postural electromyographic responses in the arm and leg following galvanic vestibular stimulation in man. Exp Brain Res 94:143–151. 10.1007/BF00230477 8335069 10.1007/BF00230477

[CR15] Burns ER, Stevens JA, Lee R (2016) The direct costs of fatal and non-fatal falls among older adults—United States. J Saf Res 58:99–103. 10.1016/j.jsr.2016.05.001 10.1016/j.jsr.2016.05.001 PMC6823838 27620939

[CR16] Carriot J, Jamali M, Cullen KE, Chacron MJ (2017) Envelope statistics of self-motion signals experienced by human subjects during everyday activities: implications for vestibular processing. PLoS ONE 12:e0178664. 10.1371/journal.pone.0178664 28575032 10.1371/journal.pone.0178664 PMC5456318

[CR17] Cathers I, Day BL, Fitzpatrick RC (2005) Otolith and Canal reflexes in human standing. J Physiol 563:229–234. 10.1113/jphysiol.2004.079525 15618274 10.1113/jphysiol.2004.079525 PMC1665570

[CR19] Curthoys IS (2017) The new vestibular stimuli: sound and vibration-anatomical, physiological and clinical evidence. Exp Brain Res 235:957–972. 10.1007/s00221-017-4874-y 28130556 10.1007/s00221-017-4874-y

[CR18] Curthoys IS, Kim J, McPhedran SK, Camp AJ (2006) Bone conducted vibration selectively activates irregular primary otolithic vestibular neurons in the Guinea pig. Exp Brain Res 175:256–267. 10.1007/s00221-006-0544-1 16761136 10.1007/s00221-006-0544-1

[CR20] Curthoys IS, MacDougall HG, Vidal P-P, de Waele C (2017) Sustained and transient vestibular systems: a physiological basis for interpreting vestibular function. Front Neurol 8:117. 10.3389/fneur.2017.00117 28424655 10.3389/fneur.2017.00117 PMC5371610

[CR21] Curthoys IS, McGarvie LA, MacDougall HG, Burgess AM, Halmagyi GM, Rey-Martinez J, Dlugaiczyk J (2023) A review of the geometric basis and the principles underlying the use and interpretation of the video head impulse test (vHIT) in clinical vestibular testing. Front Neurol 14:1147253. 10.3389/fneur.2023.1147253 37114229 10.3389/fneur.2023.1147253 PMC10126377

[CR26] Dakin CJ, Son GML, Inglis JT, Blouin J (2007) Frequency response of human vestibular reflexes characterized by stochastic stimuli. J Physiol 583:1117–1127. 10.1113/jphysiol.2007.133264 17640935 10.1113/jphysiol.2007.133264 PMC2277188

[CR25] Dakin CJ, Luu BL, Van Den Doel K et al (2010) Frequency-specific modulation of vestibular-evoked sway responses in humans. J Neurophysiol 103:1048–1056. 10.1152/jn.00881.2009 20032237 10.1152/jn.00881.2009

[CR23] Dakin CJ, Inglis JT, Blouin J-S (2011) Short and medium latency muscle responses evoked by electrical vestibular stimulation are a composite of all stimulus frequencies. Exp Brain Res 209:345–354. 10.1007/s00221-011-2549-7 21274521 10.1007/s00221-011-2549-7

[CR24] Dakin CJ, Inglis JT, Chua R, Blouin J-S (2013) Muscle-specific modulation of vestibular reflexes with increased locomotor velocity and Cadence. J Neurophysiol 110:86–94. 10.1152/jn.00843.2012 23576695 10.1152/jn.00843.2012

[CR22] Dakin CJ, Dalton BH, Luu BL, Blouin J-S (2014) Rectification is required to extract oscillatory envelope modulation from surface electromyographic signals. J Neurophysiol 112:1685–1691. 10.1152/jn.00296.2014 24990563 10.1152/jn.00296.2014

[CR27] Dalton BH, Blouin J-S, Allen MD et al (2014) The altered vestibular-evoked myogenic and whole-body postural responses in old men during standing. Exp Gerontol 60:120–128. 10.1016/j.exger.2014.09.020 25456846 10.1016/j.exger.2014.09.020

[CR28] Dietrich H, Heidger F, Schniepp R et al (2020) Head motion predictability explains activity-dependent suppression of vestibular balance control. Sci Rep 10:668. 10.1038/s41598-019-57400-z 31959778 10.1038/s41598-019-57400-z PMC6971007

[CR29] Dilda V, Morris TR, Yungher DA et al (2014) Central adaptation to repeated galvanic vestibular stimulation: implications for pre-flight astronaut training. PLoS ONE 9:e112131. 10.1371/journal.pone.0112131 25409443 10.1371/journal.pone.0112131 PMC4237321

[CR30] DiZio P, Lackner JR (1990) Age differences in oculomotor responses to step changes in body velocity and visual surround velocity. J. Gerontol Medial Sci 45:M89–94. 10.1093/geronj/45.3.m89 10.1093/geronj/45.3.m89 2335724

[CR31] Efron B, Tibshirani R (1994) An introduction to the bootstrap. Monographs on statistics and applied probability. Chapman & Hall/CRC, Boca Raton, FL

[CR32] Engstrom H, Bergstrom G, Rosenhall U (1974) Vestibular sensory epithelia. Arch Otolaryngol 100(6):411–418. 10.1001/archotol.1974.00780040425002 4217162 10.1001/archotol.1974.00780040425002

[CR33] Ernst MO, Banks MS (2002) Humans integrate visual and haptic information in a statistically optimal fashion. Nature 415:429–433. 10.1038/415429a 11807554 10.1038/415429a

[CR34] Fetsch CR, DeAngelis GC, Angelaki DE (2010) Visual-vestibular cue integration for heading perception: applications of optimal cue integration theory. Eur J Neurosci 31:1721–1729. 10.1111/j.1460-9568.2010.07207.x 20584175 10.1111/j.1460-9568.2010.07207.x PMC3108057

[CR36] Fitzpatrick RC, Day BL (2004) Probing the human vestibular system with galvanic stimulation. J Appl Physiol 96:2301–2316. 10.1152/japplphysiol.00008.2004 15133017 10.1152/japplphysiol.00008.2004

[CR35] Fitzpatrick R, Burke D, Gandevia SC (1994) Task-dependent reflex responses and movement illusions evoked by galvanic vestibular stimulation in standing humans. J Physiol 478:363–372. 10.1113/jphysiol.1994.sp020257 7965852 10.1113/jphysiol.1994.sp020257 PMC1155693

[CR37] Foulger LH, Kuo C, Chua R et al (2025) Head kinematic variability is minimal near preferred cadence and independent of the vestibular control of locomotion. Sci Rep 15:18670. 10.1152/jn.00196.2013 40437188 10.1038/s41598-025-99878-w PMC12119871

[CR38] Forbes PA, Dakin CJ, Vardy AN, Happee R, Siegmund GP, Schouten AC, Blouin J-S (2013) Frequency response of vestibular reflexes in the neck, back and lower limb muscles. J Neurophysiol 110:1869–1881. 10.1152/jn.00196.2013 23904494 10.1152/jn.00196.2013

[CR39] Forbes PA, Dakin CJ, Geers AM et al (2014) Electrical vestibular stimuli to enhance vestibulo-motor output and improve subject comfort. PLoS ONE 9:e84385. 10.1371/journal.pone.0084385 24392130 10.1371/journal.pone.0084385 PMC3879299

[CR40] Frontera WR, Hughes VA, Fielding RA, Fiatarone MA, Evans WJ, Roubenoff R (2000) Aging of skeletal muscle: a 12-yr longitudinal study. J Appl Physiol 88:1321–1326. 10.1152/jappl.2000.88.4.1321 10749826 10.1152/jappl.2000.88.4.1321

[CR41] Furman JM, Redfern MS (2001) Effect of aging on the otolith-ocular reflex. J Vestib Res 11:91–103. 10.3233/VES-2001-11204 11847453

[CR42] Grossman G, Leigh R, Abel L et al (1988) Frequency and velocity of rotational head perturbations during locomotion. Exp Brain Res 70:470–476. 10.1007/BF00247595 3384048 10.1007/BF00247595

[CR43] Ham DJ, Rüegg MA (2018) Causes and consequences of age-related changes at the neuromuscular junction. Curr Opin Physiol 4:32–39. 10.1016/j.cophys.2018.04.007

[CR44] Hannan KB, Todd MK, Pearson NJ et al (2021a) Vestibular Attenuation to random-waveform galvanic vestibular stimulation during standing and treadmill walking. Sci Rep 11:8127. 10.1038/s41598-021-87485-4 33854124 10.1038/s41598-021-87485-4 PMC8046779

[CR45] Hannan KB, Todd MK, Pearson NJ et al (2021b) Absence of nonlinear coupling between electric vestibular stimulation and evoked forces during standing balance. Front Hum Neurosci 15:631782. 10.3389/fnhum.2021.631782 33867958 10.3389/fnhum.2021.631782 PMC8046432

[CR46] Heald JB, Lengyel M, Wolpert DM (2021) Contextual inference underlies the learning of sensorimotor repertoires. Nature 600:489–493. 10.1038/s41586-021-04129-3 34819674 10.1038/s41586-021-04129-3 PMC8809113

[CR47] Hermens HJ, Freriks B, Disselhorst-Klug C, Rau G (2000) Development of recommendations for SEMG sensors and sensor placement procedures. J Electromyogr Kinesiol 10(5):361–374. 10.1016/S1050-6411(00)00027-4 11018445 10.1016/s1050-6411(00)00027-4

[CR48] Hirasaki E, Kubo T, Nozawa S et al (1993) Analysis of head and body movements of elderly people during locomotion. Acta Otolaryngol (Stockh) 113:25–30. 10.3109/00016489309126208 10.3109/00016489309126208 8447221

[CR49] Horslen BC, Dakin CJ, Inglis JT et al (2014) Modulation of human vestibular reflexes with increased postural threat. J Physiol 592:3671–3685. 10.1113/jphysiol.2014.270744 24973412 10.1113/jphysiol.2014.270744 PMC4229354

[CR109] Hullar TE, Della Santina CC, Hirvonen T, Lasker DM, Carey JP, Minor LB (2005) Responses of irregularly discharging chinchilla semicircular canal vestibular nerve afferents during high-frequency head rotations. J Neurophysiol 93(5):2777-2786. 10.1152/jn.01002.2004 10.1152/jn.01002.2004 15601735

[CR50] Iwasaki S, Smulders YE, Burgess AM et al (2008) Ocular vestibular evoked myogenic potentials to bone conducted vibration of the midline forehead at Fz in healthy subjects. Clin Neurophysiol 119:2135–2147. 10.1016/j.clinph.2008.05.028 18639490 10.1016/j.clinph.2008.05.028

[CR51] Jahn K, Naessl A, Schneider E et al (2003) Inverse U-shaped curve for age dependency of torsional eye movement responses to galvanic vestibular stimulation. Brain 126:1579–1589. 10.1093/brain/awg163 12805121 10.1093/brain/awg163

[CR52] Khosravi-Hashemi N, Forbes PA, Dakin CJ, Blouin J-S (2019) Virtual signals of head rotation induce gravity-dependent inferences. J Physiol 597:5231–5246. https://doi:10.113/JP278642 31483492 10.1113/JP278642

[CR53] Kim J (2009) Short-term habituation of eye-movement responses induced by galvanic vestibular stimulation (GVS) in the alert Guinea pig. Brain Res Bull 79:1–5. 10.1016/j.brainresbull.2008.12.016 19162141 10.1016/j.brainresbull.2008.12.016

[CR54] Kim JH, Rendon R, Von Gersdorff H (2013) Dysmyelination of auditory afferent axons increases the jitter of action potential timing during high-frequency firing. J Neurosci 33:9402–9407. 10.1523/jneurosci.3389-12.2013 23719808 10.1523/JNEUROSCI.3389-12.2013 PMC3719047

[CR55] Kirk EA, Christie AD, Knight CA, Rice CL (2021) Motor unit firing rates during constant isometric contraction: establishing and comparing an age-related pattern among muscles. J Appl Physiol 130:1903–1914. 10.1152/japplphysiol.01047.2020 33914656 10.1152/japplphysiol.01047.2020

[CR56] Kobel MJ, Wagner AR, Merfeld DM, Mattingly JK (2021) Vestibular thresholds: a review of advances and challenges in clinical applications. Front Neurol 12:643634. 10.3389/fneur.2021.643634 33679594 10.3389/fneur.2021.643634 PMC7933227

[CR108] Kwan A, Forbes PA, Mitchell DE, Blouin J-S, Cullen KE (2019) Neural Substrates, dynamics and thresholds of galvanic vestibular stimulation in the behaving primate. Nat Comm 10:1904. 10.1038/s41467-019-09738-1 10.1038/s41467-019-09738-1 PMC6478681 31015434

[CR57] Kwan KYC, Ng KWK, Rao Y, Zhu C, Qi S (2023) Effect of aging on tendon biology biomechanics and implications for treatment approaches. Int J Mol Sci 24:15183. 10.3390/ijms242015183 37894875 10.3390/ijms242015183 PMC10607611

[CR58] Lambert FM, Combes D, Simmers J, Straka H (2012) Gaze stabilization by efference copy signaling without sensory feedback during vertebrate locomotion. Curr Biol 22:1649–1658. 10.1016/j.cub.2012.07.019 22840517 10.1016/j.cub.2012.07.019

[CR59] Lee JHF, Boland-Freitas R, Ng K (2018) Sarcolemmal excitability changes in normal human aging. Muscle Nerve 57:981–988. 10.1002/mus.26058 29314071 10.1002/mus.26058

[CR60] Li C, Layman AJ, Geary R et al (2015) Epidemiology of vestibulo-ocular reflex function: data from the Baltimore longitudinal study of aging. Otol Neurotol 36:267–272. 10.1097/MAO.0000000000000610 25275869 10.1097/MAO.0000000000000610 PMC4297246

[CR61] Lim SB, Cleworth TW, Horslen BC et al (2017) Postural threat influences vestibular-evoked muscular responses. J Neurophysiol 117:604–611. 10.1152/jn.00712.2016 27832609 10.1152/jn.00712.2016 PMC5288487

[CR62] Lund S, Broberg C (1983) Effects of different head positions on postural sway in man induced by a reproducible vestibular error signal. Acta Physiol Scand 117:307–309. 10.1111/j.1748-1716.1983.tb07212.x 6603098 10.1111/j.1748-1716.1983.tb07212.x

[CR63] Luu BL, Inglis JT, Huryn TP et al (2012) Human standing is modified by an unconscious integration of congruent sensory and motor signals. J Physiol 590:5783–5794. 10.1113/jphysiol.2012.230334 22946096 10.1113/jphysiol.2012.230334 PMC3528991

[CR64] MacNeilage PR, Glasauer S (2017) Quantification of head movement predictability and implications for suppression of vestibular input during locomotion. Front Comput Neurosci 11:1–11. 10.3389/fncom.2017.00047 28638335 10.3389/fncom.2017.00047 PMC5461342

[CR65] Magnani RM, Bruijn SM, van Dieën JH (2021) Stabilization demands of walking modulate the vestibular contributions to gait. Sci Rep 11:13736. 10.1038/s41598-021-93037-7 34215780 10.1038/s41598-021-93037-7 PMC8253745

[CR66] Maris E, Oostenveld R (2007) Nonparametric statistical testing of EEG- and MEG-data. J Neurosci Methods 164:177–190. 10.1016/j.jneumeth.2007.03.024 17517438 10.1016/j.jneumeth.2007.03.024

[CR67] McFadyen BJ, Winter GA (1988) An integrated biomechanical analysis of normal stair ascent and descent. J Biomech 21:733–744 3182877 10.1016/0021-9290(88)90282-5

[CR68] Merchant SN, Veläzquez-Villasenor L, Tsuji K, Glynn RJ (2000) Temporal bone studies of the human peripheral vestibular system. Rhinol Laryngol 109:3–13. 10.1177/00034894001090s502 10.1177/00034894001090s502 10821229

[CR69] Neiman AB, Russel DF, Rowe MH, Zochowski M (2011) Identifying Temporal codes in spontaneously active sensory neurons. PLoS ONE 6:e27380. 10.1371/journal.pone.0027380 22087303 10.1371/journal.pone.0027380 PMC3210806

[CR70] Nguyen KD, Welgampola MS, Carey JP (2010) Test-retest reliability and age-related characteristics of the ocular and cervical vestibular evoked myogenic potential tests. Otol Neurotol 31:793–802. 10.1097/MAO.0b013e3181e3d60e 20517167 10.1097/MAO.0b013e3181e3d60e PMC2913294

[CR71] Ochi K, Ohashi T (2003) Age-related changes in the vestibular-evoked myogenic potentials. Otolaryngol-Head Neck Surg 129:655–659. 10.1016/s0194-5998(03)01578-x 14663431 10.1016/s0194-5998(03)01578-x

[CR72] Paige GD (1991) The aging vestibulo-ocular reflex (VOR) and adaptive plasticity. Acta Otolaryngol (Stockh) 111 Sup 481:297–300. 10.3109/00016489109131406 10.3109/00016489109131406 1927400

[CR75] Peterka RJ (2002) Sensorimotor integration in human postural control. J Neurophysiol 88:1097–1118. 10.1152/jn.00605.2001 12205132 10.1152/jn.2002.88.3.1097

[CR74] Peterka RJ, Black FO, Schoenhoff MB (1990) Age-related changes in human vestibulo-ocular reflexes: sinusoidal rotation and caloric tests. J Vestib Res 1:49–59. 10.3233/VES-1990-1106 1670137

[CR76] Peters RM, Blouin J-S, Dalton BH, Inglis JT (2016) Older adults demonstrate superior vestibular perception for virtual rotations. Exp Gerontol 82:50–57. 10.1016/j.exger.2016.05.014 27262689 10.1016/j.exger.2016.05.014

[CR77] Phillips C, Shepherd SJ, Nowack A et al (2016) Loss of afferent vestibular input produces central adaptation and increased gain of vestibular prosthetic stimulation. J Assoc Res Otolaryngol 17:19–35. 10.1007/s10162-015-0544-6 26438271 10.1007/s10162-015-0544-6 PMC4722019

[CR73] Piasecki M, Ireland A, Jones DA, McPhee JS (2016) Age-dependent motor unit remodeling in human limb muscles. Biogerontology 17:485–496. 10.1007/s10522-015-9627-3 26667009 10.1007/s10522-015-9627-3 PMC4889636

[CR79] Piker EG, Jacobson GP, McCaslin DL, Hood LJ (2011) Normal characteristics of the ocular vestibular evoked myogenic potential. J Am Acad Audiol 22:222–230. 10.3766/jaaa.22.4.5 21586257 10.3766/jaaa.22.4.5

[CR78] Piker EG, Jacobson GP, Burkard RF et al (2013) Effects of age on the tuning of the cVEMP and oVEMP. Ear Hear 34:e65–e73. 10.1097/AUD.0b013e31828fc9f2 23673615 10.1097/AUD.0b013e31828fc9f2 PMC3748259

[CR80] Pozzo T, Berthoz A, Lefort L (1990) Head stabilization during various locomotor tasks in humans. I. Normal subjects. Exp Brain Res 82:97–106. 10.1007/BF00230842 2257917 10.1007/BF00230842

[CR81] Raj IS, Bird SR, Shield AJ (2010) Aging and the force-velocity relationship of muscles. Exp Gerontol 45:81–90. 10.1016/j.exger.2009.10.013 19883746 10.1016/j.exger.2009.10.013

[CR82] Reeves ND, Spanjaard M, Mohagheghi AA, Baltzopoulos V, Maganaris CN (2008) The demands of stair descent relative to maximum capacities in elderly and young adults. J Electromyogr Kinesiol 18:218–227. 10.1016/j.jelekin.2007.06.003 17822923 10.1016/j.jelekin.2007.06.003

[CR83] Richter E (1980) Quantitative study of human scarpa’s ganglion and vestibular sensory epithelia. Acta Otolaryngol (Stockh) 90:199–208. 10.3109/00016488009131716 6258381 10.3109/00016488009131716

[CR84] Roditi RE, Crane BT (2012) Directional asymmetries and age effects in human self-motion perception. J Assoc Res Otolaryngol 13:381–401. 10.1007/s10162-012-0318-3 22402987 10.1007/s10162-012-0318-3 PMC3346890

[CR85] Rosengren SM, Govender S, Colebatch JG (2011) Ocular and cervical vestibular evoked myogenic potentials produced by air- and bone-conducted stimuli: comparative properties and effects of age. Clin Neurophysiol 122:2282–2289. 10.1016/j.clinph.2011.04.001 21550301 10.1016/j.clinph.2011.04.001

[CR86] Rosenhall U (1973) Degenerative patterns in the aging human vestibular neuro-epithelia. Acta Otolaryngol 76:208–220. 10.3109/00016487309121501 4543916 10.3109/00016487309121501

[CR87] Rubinstein S, Kamen G (2005) Decreases in motor unit firing rate during sustained maximal-effort contractions in young and older adults. J Electromyogr Kinesiol 15:536–543. 10.1016/j.jelekin.2005.04.001 16054395 10.1016/j.jelekin.2005.04.001

[CR88] Samuel D, Rowe P, Hood V, Nicol A (2011) The Biomechanical functional demand placed on the knee and hip muscles of older adults during stair ascent and descent. Gait Pasture 34:239–244. 10.1016/j.gaitpost.2011.05.005 10.1016/j.gaitpost.2011.05.005 21632255

[CR89] Schneider AD, Jamali M, Carriot J, Chacron MJ, Cullen KE (2015) The increased sensitivity of irregular peripheral Canal and otolith afferents optimizes their encoding of natural stimuli. J Neurosci 35:5522–5536. 10.1523/JNEUROSCI.3841-14.2015 25855169 10.1523/JNEUROSCI.3841-14.2015 PMC4388918

[CR90] Seemungal BM, Gunaratne IA, Fleming IO et al (2004) Perceptual and nystagmic thresholds of vestibular function in Yaw. J Vestib Res 14:461–466. 10.3233/VES-2004-14604 15735328

[CR91] Sperry RW (1950) Neural basis of the spontaneous optokinetic response produced by visual inversion. J Comp Physiol Psychol 43:482–489. 10.1037/h0055479 14794830 10.1037/h0055479

[CR92] Stefansson S, Imoto T (1986) Age-related changes in optokinetic and rotational tests. Am J Otol 7:193–196 3487250

[CR93] Su H-C, Huang T-W, Young Y-H, Cheng P-W (2004) Aging effect on vestibular evoked myogenic potential. Otol Neurotol 25:977–980. 10.1097/00129492-200411000-00019 15547429 10.1097/00129492-200411000-00019

[CR94] Teh KC, Aziz AR (2002) Heart rate, oxygen uptake and energy cost of ascending and descending the stairs. Med Sci Sports Exerc 34:695–699 11932581 10.1097/00005768-200204000-00021

[CR95] Tseng C, Chou C, Young Y (2010) Aging effect on the ocular vestibular-evoked myogenic potentials. Otol Neurotol 31:959–963. 10.1097/MAO.0b013e3181e8fb1a 20601917 10.1097/MAO.0b013e3181e8fb1a

[CR96] Verdú E, Ceballos D, Vilches JJ, Navarro X (2000) Influence of aging on peripheral nerve function and regeneration. JPNS 5:191–208. 10.111/j.1529-8027.2000.00026.x 11151980

[CR97] Von Holst E, Mittelstaedt H (1950) The reafference principle–interactions between the central nervous system and the periphery. Naturwissenschaften. 37:464–476. 10.1007/BF00622503

[CR98] Von Uckermann G, Le Ray D, Combes D, Straka H, Simmers J (2013) Spinal efference copy signaling and gaze stabilization during locomotion in juvenile xenopus frogs. J Neurosci 33(10):4253–4264. 10.1523/JNEUROSCI.4521-12.2013 23467343 10.1523/JNEUROSCI.4521-12.2013 PMC6704964

[CR99] Wagner AR, Akinsola O, Chaudhari AMW, Bigelow KE, Merfeld DM (2021) Measuring vestibular contributions to age-related balance impairment: a review. Front Neurol 12:635305. 10.3389/fneur.2021.635305 33633678 10.3389/fneur.2021.635305 PMC7900546

[CR100] Wall C, Black F, Hunt A (1984) Effects of age, sex and stimulus parameters upon vestibulo-ocular responses to sinusoidal rotation. Acta Otolaryngol (Stockh) 98:270–278. 10.3109/00016488409107563 6333770 10.3109/00016488409107563

[CR101] Wiesmeier IK, Dalin D, Maurer C (2015) Elderly use proprioception rather than visual and vestibular cues for postural motor control. Front Aging Neurosci 7:97. 10.3389/fnagi.2015.00097 26157386 10.3389/fnagi.2015.00097 PMC4477145

[CR103] Welgampola MS, Colebatch JG (2001) Vestibulocollic reflexes: normal values and the effect of age. Clin Neurophysiol 112:1971–1979. 10.1016/S1388-2457(01)00645-9 11682335 10.1016/s1388-2457(01)00645-9

[CR102] Welgampola M, Colebatch J (2002) Selective effects of ageing on vestibular-dependent lower limb responses following galvanic stimulation. Clin Neurophysiol 113:528–534. 10.1016/S1388-2457(02)00020-2 11955997 10.1016/s1388-2457(02)00020-2

[CR104] Wu R, Ditroilo M, Delahunt E, De Vito G (2021) Age related changes in motor function (II) decline in motor performance outcomes. Int J Sports Med 42:215–226. 10.1055/a-1265-7073 33137831 10.1055/a-1265-7073

[CR105] Zalewski C (2015) Aging of the human vestibular system. Semin Hear 36:175–196. 10.1055/s-0035-1555120 27516717 10.1055/s-0035-1555120 PMC4906308

[CR106] Zapala DA, Brey RH (2004) Clinical experience with the vestibular evoked myogenic potential. J Am Acad Audiol 15:198–215. 10.3766/jaaa.15.3.3 15119461 10.3766/jaaa.15.3.3

[CR107] Zhan Y, Halliday D, Jiang P et al (2006) Detecting time-dependent coherence between non-stationary electrophysiological signals—a combined statistical and time–frequency approach. J Neurosci Methods 156:322–332. 10.1016/j.jneumeth.2006.02.013 16563517 10.1016/j.jneumeth.2006.02.013

